# Layer-by-Layer Assembly of Biopolyelectrolytes onto Thermo/pH-Responsive Micro/Nano-Gels

**DOI:** 10.3390/ma7117472

**Published:** 2014-11-21

**Authors:** Ana M. Díez-Pascual, Peter S. Shuttleworth

**Affiliations:** 1 Analytical Chemistry, Physical Chemistry and Chemical Engineering Department, Faculty of Biology, Environmental Sciences and Chemistry, Alcalá University, 28871 Alcalá de Henares, Madrid, Spain; 2 Instituto de Ciencia y Tecnología de Polímeros-Consejo Superior de Investigaciones Científicas, Juan de la Cierva 3, 28006 Madrid, Spain; E-Mail: peter@ictp.csic.es

**Keywords:** layer-by-layer, thermoresponsive gels, polyelectrolytes, polypeptides, polysaccharides, biomedical applications

## Abstract

This review deals with the layer-by-layer (LbL) assembly of polyelectrolyte multilayers of biopolymers, polypeptides (*i.e.*, poly-l-lysine/poly-l-glutamic acid) and polysaccharides (*i.e.*, chitosan/dextran sulphate/sodium alginate), onto thermo- and/or pH-responsive micro- and nano-gels such as those based on synthetic poly(*N-*isopropylacrylamide) (PNIPAM) and poly(acrylic acid) (PAA) or biodegradable hyaluronic acid (HA) and dextran-hydroxyethyl methacrylate (DEX-HEMA). The synthesis of the ensembles and their characterization by way of various techniques is described. The morphology, hydrodynamic size, surface charge density, bilayer thickness, stability over time and mechanical properties of the systems are discussed. Further, the mechanisms of interaction between biopolymers and gels are analysed. Results demonstrate that the structure and properties of biocompatible multilayer films can be finely tuned by confinement onto stimuli-responsive gels, which thus provides new perspectives for biomedical applications, particularly in the controlled release of biomolecules, bio-sensors, gene delivery, tissue engineering and storage.

## 1. Introduction

The use of oppositely charged polyelectrolytes (PEs) on a charged surface to build controlled architecture multilayer polymer films based on layer-by-layer (LbL) assembly was first reported in 1992 by Decher* et al.* [1,2]. The use of flat and rigid structures was originally applied for the construction of multilayered PEs, with parameters such as, PE composition and conformation, charge density, pH, ionic strength and investigated temperature [3,4]. PEs can be either synthetic or natural and can be further classified into strong or weak depending whether they are fully or partially charged. Typically, multilayers were prepared from strong PEs like poly(diallyldimethylammonium chloride) (PDACMAC) or poly(sodium 4-styrenesulfonate) (PSS) that remain fully charged over the entire pH range, and the addition of salt was the primary means to control the thickness of the adsorbed layers [1]. However, this approach is limited to a certain extent by the poor solubility of high molecular weight PEs in solutions of high ionic strength. To overcome this limitation and make the LbL adsorption process more versatile, weak PEs like synthetic poly(allylamine hydrochloride) (PAH) and poly(acrylic acid) (PAA) [5], as well as natural polysaccharides [6,7,8] and polypeptides [8,9,10] that are capable of accessing a very diverse range of configurations were explored. In these cases, it was possible to systematically vary the linear charge density of the polymer by simply adjusting the pH of the dipping solutions [11]. When a weak PE is incorporated into a multilayer system, its degree of ionization may change considerably from the solution value due to the influence of the local environment through electrostatic and hydrophobic effects. Electrostatic effects [11] are observed upon addition of salt to the PE solutions or when an oppositely charged polymer is added to form a complex. Hydrophobic effects occur when the PE experiences hydrophobic moieties or regions that can alter the dielectric environment of the weak ionic group, therefore making it more difficult to achieve an ionized state. Due to the mentioned effects, the pK_a_ of the PE in the multilayer (pH at which 50% of the polymer functional groups are ionized) depends strongly on the nature of the other polymer used in the assembly process.

Besides PEs, the LbL technique has also been extended to other organic and inorganic structural forms by Donath* et al.* [12] and Caruso* et al.* [13]. Through use of the LbL techniques, PEs were deposited on sacrificial spherical core particles, which were then subsequently removed leaving a residual hollow capsule. In addition, the same authors made interconnecting networks from various PE complexes via coating of both hard and porous templates like mesoporous silica [14] or CaCO_3_ [15]. This approach can be adopted for the encapsulation of high loadings of therapeutics due to the porous material’s high surface area and pore volume, and is applicable to a wide range of compounds of different sizes, from proteins to low molecular weight drugs. A more recent approach is to carry out the LbL assembly on stimuli-responsive gels, a class of “smart” materials that have the ability to adapt and respond to external stimuli such as pH, temperature, ionic strength, light, electric or magnetic field, chemical or biological compounds, and consequently have a wide range of applications that include sensors, drug delivery, gene delivery, medical devices and tissue engineering [16,17]. In particular, sensitive microgels (MGs) and nanogels (NGs) are highly interesting since they exhibit exceptional properties arising from the combination of their colloidal nature (colloidal stability, high surface area, facile synthesis and control over particle size) with their internal network structure [18]. The surface modification of MGs (or NGs) via the LbL approach can result in assembled core-shell structures with new thermo/pH-responsive properties that offer an attractive means for encapsulation/immobilization (storage) and delivery of a variety of substances, especially dyes, proteins and drug moieties. Thus, the gel properties can be tailored and their stability improved by depositing a PE shell, which can govern the transport of substances into and out of the resulting core-shell ensemble. With the proper choice of PE pairs, selective permeability can be achieved as well as a sustained release of a variety of substances. The MG (or NG) level of porosity plays a key role in the LbL assembly since the adsorbing PE layers can not only interdigitate among themselves, as when dealing with hard and rigid templates, but also penetrate into the gel, conferring novel surface properties. The extent of interpenetration is conditioned by the mesh size of the polymeric network (associated to the degree of cross-linking), and also by the PE molecular weight and degree of branching [19]. The larger the pore size, the easier the movement of the PE within the MG/NG. Further, a highly branched or a high molecular weight PE is expected to have a lower degree of interpenetration with the gel. Although the PE adsorption process is mainly electrostatically-driven, several secondary cooperative interactions such as hydrogen bonding, van der Waals forces and hydrophobic interactions are also important to construct multilayers, especially when dealing with weak PEs and other materials [20,21]. The motor of the growth is provided by the charge overcompensation that appears after each deposition step and is required for the multilayer build up, mainly during the adsorption of the first layers of the multilayer films, which is evidenced by the alternation of the zeta potential during its build up [8].

This review focuses on the LbL assembly of different biopolymers, namely polypeptides (*i.e.*, poly-l-lysine (PLL), poly-l-glutamic acid (PGA), poly-l-arginine (pARG) and poly-l-aspartic acid (pASP)) and polysaccharides (*i.e.*, chitosan (CHIT), dextran sulphate (DEXS), and sodium alginate (ALG)) onto thermo- and/or pH-responsive MGs (or NGs), like those based on synthetic poly(*N-*isopropylacrylamide) (PNIPAM) and poly(acrylic acid) (PAA) or biodegradable hyaluronic acid (HA) and dextran-hydroxyethyl methacrylate (DEX-HEMA). Natural polypeptides and polysaccharides are in general weak PEs and can adopt multiple conformations in response to changes in the solution pH and temperature. The folding and unfolding of their chains through an optimal balance via hydrogen bonds, hydrophobic, electrostatic, and other interactions provide fascinating properties for natural PEs to interact with MGs (or NGs) via LbL assembly. Selected examples have been presented to demonstrate how subtle changes in the internal reorganization of the gels during heating and/or cooling can induce or restrict, through various types of non-covalent interactions, conformational changes in the absorbed PE layers; likewise, to show how these processing parameters affect the structure and stability of the multilayer films as well as their kinetics of folding in a confined geometry. In addition, the influence of the number of layers, the order by which the layers are adsorbed and the nature of the last layer adsorbed on the morphology, the thermoresponsive behaviour, the surface charge density, the bilayer thickness, the temporal stability and the mechanical properties of coated MGs (or NGs) are described. Further, the interactions between the gels and the PEs are analysed. The approaches described herein integrate gel structures with biopolymers, thus constituting simple, cost-effective, and highly-versatile methods to develop multifunctional materials suitable for myriad applications, including biomedical and biotechnological applications.

## 2. Micro/Nano-Gels: Structure and Properties

A gel is a 3D cross-linked polymeric network swollen by a solvent (lyogel) or by a gas (aerogel). The most common are lyogels in which the solvent is water, also called “*hydrogels*”. Particularly attractive are those having a responsive character, which responds to external stimuli with a change in size. Macrogels react relatively slowly to these external parameters due to their macroscopic dimensions. In 1935, Staudinger and Husemann [22] observed that the crosslinking polymerization of a dilute solution of divinylbenzene led to a soluble polymer of low viscosity consisting of strongly branched 3D molecules. These inter- and intra-molecularly crosslinked globular macromolecules dispersed in either normal or colloidal solutions are named as “*microgels*, *hydrogel nanoparticles* or *nanogels*”. They have colloidal dimensions with diameters in the swollen state ranging from 100 nm up to 5 μm [18] and can respond almost spontaneously to changes of external parameters. It should be noted that no clear correlation between the size of the gel particles and the nomenclature has been established and the terms “*microgel*” and “*nanogel*” are frequently used interchangeably in the literature. MGs with a controlled size can be synthesized in a homogeneous phase via free radical crosslinking copolymerization of mono- and bis-unsaturated monomers in dilute solutions or through the heterophase copolymerization of monomers with crosslinking agents in aqueous solution by either precipitation polymerization, crosslinking of preformed polymers in inverse water-in-oil emulsions or microemulsions [18].

### 2.1. Synthetic Micro/Nano-Gels

**Scheme 1:**
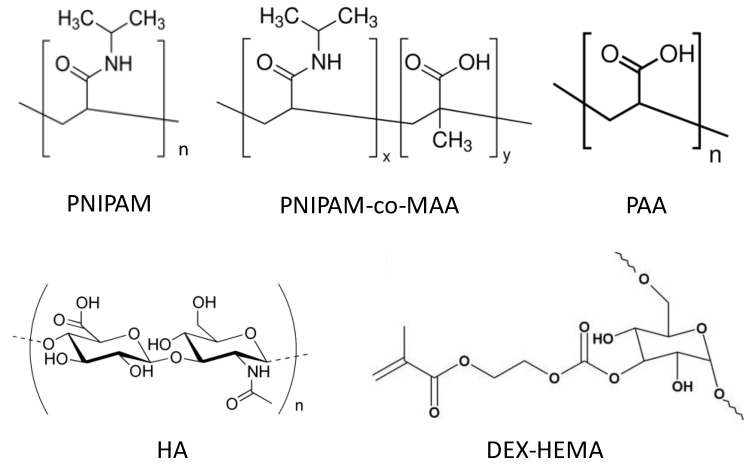
Chemical structures of poly(*N-*isopropylacrylamide) (PNIPAM), PNIPAM-*co*-methacrylic acid (PNIPAM-*co*-MAA), poly(acrylic acid) (PAA), hyaluronic acid (HA) and dextran-hydroxyethyl methacrylate (DEX-HEMA).

The most common thermosensitive microgels are those based on PNIPAM (Scheme 1) [18], which present a lower critical solution temperature (LCST) in water. The phenomenon consists in an entropically driven phase transition from a swollen to a collapsed state at temperatures above 32 °C [23]; a temperature useful for biomedical applications since it is close to that of the human body (37 °C). Two types of interactions have to be considered to understand this behaviour: hydrogen bonding between the amide groups and water and polymer-polymer interactions due to the hydrophobic isopropyl groups. At low temperatures the formation of H-bonds between the amide groups of PNIPAM and the water molecules are thermodynamically favourable, and the polymer displays a hydrophilic random coil configuration. Nonetheless, increasing the temperature above the LCST leads to the prevalence of hydrophobic interactions, in which water is expulsed due to the weakened hydrogen bonds and the polymer chains collapse into a globule. In the case of MGs formed from crosslinked PNIPAM networks, the thermosensitivity is transferred to the whole system. Thus, at temperatures below the LCST of the polymer, the gel nanoparticles are swollen, but they shrink with increasing temperature. This transition takes place at the volume phase transition temperature (VPTT) which is close to the LCST of the PNIPAM chains. A slight shift in the VPTT has been observed by copolymerizing with hydrophilic or hydrophobic monomers [18]; for instance, the VPTT of PNIPAM-co-methacrylic acid (PNIPAM-*co*-MAA) was reported to be around 33 °C due to specific H-bonding [8]. In these microgels (Scheme 1), the main monomer, NIPAM, provides thermosensitivity to the network while the comonomer, methacrylic acid, enables pH tuneability, thus modifying the charge and swelling behaviour. Other polymers with thermoresponsive character are poly(*N*-vinlycaprolactam) (PVCL) with LCST in the range of 25–35 °C, poly(*N,N-*diethylacrylamide) (PDEAAM) with LCST between 25 and 32 °C, poly[*2*-(dimethylamino)ethyl methacrylate] (PDMAEMA) with LCST of ~50 °C and poly(ethylene glycol) (PEG) in which the LCST takes place at 85 °C [16]. Nevertheless, the LCST is strongly affected by the polymer molecular weight and architecture.

There are two types of pH-sensitive microgels: those containing pendant acidic groups (*i.e.*, COOH, SO_3_H) that become ionized at basic pH, and others incorporating basic groups (NH_2_) that acquire charge in acidic pH. Poly(acrylic acid) (PAA) is an example of the former type (see Scheme 1), while poly (*N*,*N*-diethylaminoethyl methaacrylate) (PDEAEM) belongs to the second class. The mechanism of response is the same for both; when the polymer chains are charged there is an electrostatic repulsion that causes the microgel to expand, while when the functional groups lose their charge the repulsion disappears and the material collapses again.

### 2.2. Naturally Derived Micro/Nano-Gels

Microgels derived from naturally occurring polysaccharides such as hyaluronic acid (HA) have recently been the focus of considerable research due to their high biocompatibility and biodegradability, low toxicity, abundance in nature, and low cost [24]. HA is a linear anionic polysaccharide composed of repeating units of d-glucuronic acid and *N*-acetyl-d-glucosamine (Scheme 1), and can be found in connective, epithelial, and neural tissues. The pK_a_ of its COOH groups is 3–4; thus, at physiological conditions these moieties are ionized, and HA adopts an expanded random coil structure. HA is a highly hydrophilic polymer that can absorb a large amount of water and expand up to 1000 times its solid volume, forming a loose hydrated network [24]. However, pristine HA presents some drawbacks that limit its use for practical applications, such as its susceptibility to degradation and poor mechanical properties. To afford HA-based hydrogels with enhanced mechanical properties and degradation rates and simultaneously maintain their native biological functions, controlled chemical modification and covalent crosslinking are typically required. By varying the HA molecular weight, the degree of modification and the concentration of its reactive precursors, hydrogels with varying stiffness, pore size and degradation rate can be obtained.

Dextran (DEX) is another polysaccharide derivative widely employed for the preparation of hydrogels for controlled protein release. It can be produced thermally, chemically or from bacterial processing of sucrose. Bacterial dextran consists of linear 1,6-linked glucopyranose units with some degree of branching. DEX has been modified with different molecules like hydroxethylmethacrylate (HEMA) for the formation of chemically crosslinked DEX-HEMA microgels (Scheme 1) [6]. These gels are biodegradable via hydrolysis of the carbonate esters in the crosslinks that connect the dextran chains. Upon cleavage of the crosslinks, the swelling pressure of the microgels increases, and may result in rupture of the surrounding membrane, depending on the properties of the membrane and the pH of the environment. It has been demonstrated [6,25] that self-rupturing microcapsules can be obtained when DEX-HEMA microgels are coated with PEs and incubated at physiological pH, therefore showing great potential as a pulsed drug delivery system.

## 3. Biopolyelectrolytes Used for the LbL Assembly: Structure and Properties

### 3.1. Synthetic Biopolyelectrolytes

**Scheme 2:**
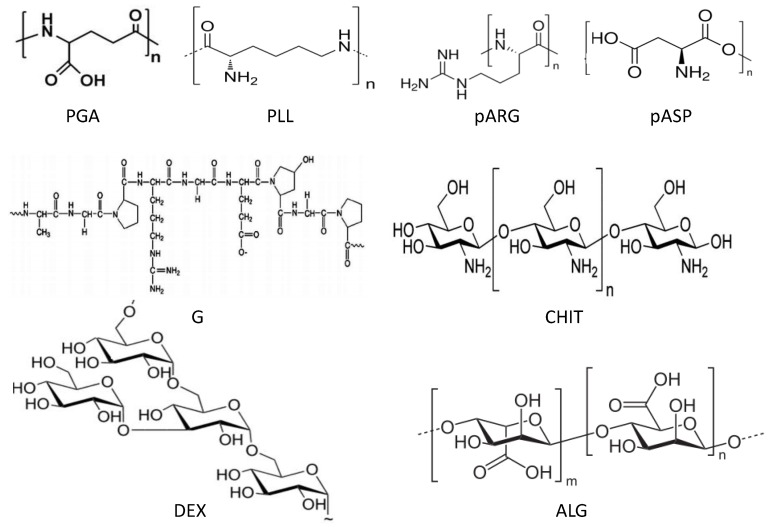
Schematic representation of the polypeptides and polysaccharides used for the layer-by-layer (LbL) process: poly-l-glutamic acid (PGA), poly-l-lysine (PLL), poly-l-arginine (pARG), poly-l-aspartic acid (pASP), gelatine (G), chitosan (CHIT), dextran (DEX) and alginate (ALG).

Polypeptides such as poly-l-lysine (PLL) and poly-l-glutamic acid (PGA) are valuable model systems for studying protein conformational changes since they can adopt multiple conformations such as α-helix, β-sheet or random coil structures in response to changes in the pH and temperature conditions (Scheme 2). Both PLL and PGA are weak PEs (pK_a_ ~10.5 and 4.3, respectively), and become highly charged at neutral pH, in which they tend to adopt a random coil conformation. The amino groups of PLL are neutralized at high pH (~11) and the polypeptide adopts an α-helical configuration. Further, heating a PLL solution with low salt concentration for a short period of time converts the unordered random coil state to a folded β-sheet structure, which is retained after the solution is cooled to ambient temperature [8]. In the PLL/PGA system, complexes in solution are expected to form β-sheet structures in antiparallel orientation within sheets and parallel orientation between sheets. Although strong electrostatic interactions are dominant, hydrogen bonding and hydrophobic interactions also play a key role in the LbL assembly.

Poly-l-arginine (pARG) and poly-l-aspartic acid (pASP) are also biodegradable polyaminoacids (Scheme 2). pARG is currently used for the treatment of head and neck cancer; it is the only polypeptide with side groups that are charged at almost every pH (the pK_a_ of the side groups is 12.5), and is therefore a strong polycation. It adopts a random coil structure in the pH range from 2.7 to 11, although it changes to an α-helical configuration at higher pH. pASP is an environmentally friendly, biodegradable alternative to traditional polyanionic materials like PAA and can be used as a super-swelling material in nappy/hygiene products and food packaging. It has a pK_a_ of ~3.9, and can adopt random-coil and β-antiparallel pleated-sheet conformations in both acidic and basic conditions, while the α-helical conformation appears only at low pH.

Gelatine (G) is a heterogeneous mixture of protein fractions consisting of single or multi-stranded polypeptides, each with an extended left-handed proline helix conformation, containing between 50 and 1000 amino acids. It is obtained by the partial hydrolysis of animal collagen derived from skin, white connective tissues and bones. G contains mainly glycine, proline, 4-hydroxyproline, glutamic acid, alanine, arginine and aspartic acid residues, hence, it possesses both positive and negative charges. The denaturalization process through which G is obtained can be performed by acid hydrolysis, leading to gelatine A with pK_a_ of ~6, or alkaline hydrolysis resulting in gelatine B with pK_a_ of 4.7. Gelatine A shows cationic behaviour at pH below its pK_a_ due to protonation of amino groups, while Gelatine B is negatively charge at pH values above 4.7.

### 3.2. Naturally Derived Biopolyelectrolytes

Chitosan (CHIT) and dextran sulfate (DEXS) are interesting biocompatible and biodegradable polysaccharides for medical use, since their alternate assembly exhibits anti* versus* pro-coagulant activity of human whole blood. Chitosan refers to a series of polymers that are deacetylated derivatives of the natural polysaccharide, chitin, with different degrees of deacetylation (typically between 70% and 95%) and molecular weights. It is a linear polycation composed of β-1,4-linked glucosamine (deacetylated units) and *N*-acetyl-d-glucosamine (acetylated units), with a pK_a_ of ~6.0, hence is positively charged in strong acid conditions (Scheme 2). DEXS is a branched polyanion produced by esterification of dextran with chlorosulphonic acid, and possesses a carbohydrate backbone incorporating about 2.3 negatively charged sulphate groups per glucosyl residue, with a pK_a_ of ~4.0. Alginate (ALG) is another biodegradable polysaccharide consisting of 1,4-linked β-d-mannuronic acid and α-L-guluronic acid monomers in varying proportions (Scheme 2). It is a linear polyanion with pK_a_ of about 3.2, and is commonly used to form polyelectrolyte complexes with CHIT because these are also biodegradable and biocompatible, but mechanically stronger than the neat biopolymers. All these polysaccharides are weak PEs, and just like polypeptides, present different structures, such as various helical and ribbon-like secondary structures. When assembled in a multilayer structure, their relevant interactions are hydrogen bonding, dipole and ionic interactions, and solvation terms. Conformational stability is usually achieved through secondary cooperative interactions between two or more chain segments, usually with long regions of regular covalence sequence. When such regions are interrupted by chain branching or other altered substitution, several chain associations can give rise to higher levels of structure and result in a three-dimensional network.

## 4. Synthesis of Micro/Nano-Gels and Preparation of Biopolymer-Coated Gels

### 4.1. Synthesis of PNIPAM-Based Hydrogels

P(NIPAM-*co*-MAA) NGs have been synthesized by free radical emulsion polymerization [8,26]. The NIPAM monomer, the cross-linker *N,N*'-methylenebis(acrylamide) and the co-monomer methacrylic acid (MAA) (9:0.5:1 ratio) were dissolved in water at 70 °C and purged with nitrogen for 40 min while stirring. Polymerization was initiated with a water solution of potassium peroxodisulfate (KPS) and allowed to proceed for about 6 h under a nitrogen stream and constant stirring. The dispersion was passed through glass wool in order to remove particulate matter and further purified by dialysis against deionized and double-distilled water for 1 week. Then the solution was centrifuged for several cycles; between each centrifugation cycle and the supernatant was removed and replaced by deionised water to re-disperse. Finally the solution was lyophilized (freeze-dried) overnight for storage. The produced NGs had an average size of 300 nm.

Following a similar procedure, P(NIPAM-*co*-MAA) MGs were recently synthesized in supercritical carbon dioxide (scCO_2_) [27]. The monomers (5.2 wt% content relative to CO_2_) and perfluoropolyether (10 wt% content relative to the monomers) were charged into a high pressure cell at 28.0 MPa and 65 °C. All the reactants were soluble in the supercritical medium, and the reaction was allowed to proceed for 24 h under stirring. The obtained polymer was washed continuously with fresh CO_2_ for 1 h to remove any residual monomers or cross-linker. Monodisperse cross-linked MGs with an average diameter of 3.0 ± 0.7 μm were subsequently obtained.

### 4.2. Preparation of PAA Microgels

PAA microgels were synthesized by inverse suspension polymerization using cyclohexane as the continuous phase and sorbitan monostearate as nonionic surfactant [9,10]. Firstly, the surfactant was dissolved in cyclohexane and the solution heated to 45 °C in a nitrogen atmosphere. A mixture of acrylic acid, *N,N*'-methylenebis(acrylamide), NaOH, NaCl, *N*,*N*,*N*',*N*'-tetramethylethylenediamine (TEMED) and ammonium persulfate solution was then added to the initial mixture. The polymerization reaction was carried out at 65 °C for 30 min in a nitrogen atmosphere, and the reaction was stopped by addition of methanol. Gel particles were repeatedly washed with methanol followed by equilibration with purified water. Monodisperse MGs with particle size ≤300 μm were collected.

### 4.3. Preparation of HA-Based Microgels

Thiolated HA was prepared by reacting HA with cystamine in the presence of [1-ethyl-3-(dimethylamino)propyl]carbodiimide hydrochloride solution in phosphate buffered saline [28]. The cystamine conjugated HA was then treated with dithiothreitol, dialyzed against deionized water for 2 days, and lyophilized. The degree of HA functionalization was about 40%. Subsequently, a solution of thiolated HA was added to hexane under stirring to produce a water-in-oil inverse suspension, H_2_O_2_ was added and the mixture was stirred for 3 h. Cross-linked HA MGs with an average diameter of 16.2 μm were collected by centrifugation and washed three times with a mixture of water/acetone.

Vinylbenzene(VB)-modified HA derivatives were synthesized by grafting different amounts of VB onto the carboxylic acid groups of HA via an esterification reaction [29]. HA salt (in its acid form) and VB were dissolved in dimethyl sulfoxide (DMSO) at 40 °C, and the reaction was continued under magnetic stirring for 48 h. Then, a concentrated aqueous solution of NaCl was added to change the obtained VB-grafted HA into the sodium salt form that was precipitated in acetone. The precipitate was washed with acetone, purified by dialysis against water for 6 days and lyophilized.

Hydrazide-modified HA derivatives were prepared by adding adipic dihydrazide (ADH) to a solution of HA in water [30]. Then, an aqueous solution of 1-ethyl-3-[3-(dimethylamino)propyl] carbodiimide (EDC) was added to the mixture and the reaction was allowed to proceed at room temperature for 4 h. The modified HA was purified through an ultra-filtration membrane and lyophilized. Different alkylamino derivatives were then prepared by adding aldehydic chains (1-hexanal, 1-octanal or 1-decanal) to a solution of hydrazide-modified HA in ethanol, followed by a solution of 2-picoline borane complex. After stirring for 24 h at room temperature, the modified HA was precipitated with EtOH, successively washed with different mixtures of EtOH/H_2_O and finally filtered. HA derivatives with alkyl chains of 6–10 carbon atoms and degrees of substitution (DS) per disaccharide repeat unit ranging from 0.05 to 0.1 were obtained.

### 4.4. Preparation of DEX-HEMA Microgels

Biodegradable DEX-HEMA MGs can be prepared by an aqueous emulsion technique: DEX-HEMA, fluorescein isothiocyanate (FITC)-labelled DEX solution and dimethyl aminoethyl methacrylate (DMAEMA) were dissolved in water, and subsequently emulsified with an aqueous poly(ethylene glycol) (PEG) solution [6]. Radical polymerization of the pending HEMA groups was initiated by adding TEMED and KPS. The reaction was carried out at room temperature for 1 h. Finally, the synthesized MGs (with an average diameter of 7 μm) were washed three times with pure water to remove PEG, KPS, and TEMED, suspended in water and stored at −20 °C.

### 4.5. Layer-by-Layer Assembly of Biopolyelectrolytes

**Scheme 3:**
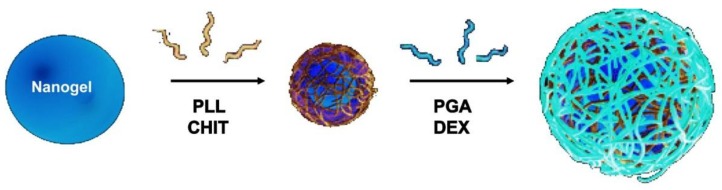
Schematic representation of the LbL assembly of biopolymers onto soft and porous nanogels (NGs). Reproduced with permission from [8], copyright 2010, Elsevier.

The multilayer assembly of PNIPAM-*co*-MAA was performed by adding an aqueous dispersion of the gel (0.02–0.05 wt%) to 1 mg/mL PE solution [8,27]. PLL and PGA solutions were prepared in buffer (25 mM Tris-(hydroxymethylaminomethane), 10 mM 2-(*N*-morpholino)-ethanesulfonic acid and 1.15 M NaCl, pH = 7.5). At neutral pH, both PLL and PGA are ionized. CHIT was dissolved in an aqueous solution with 25% acetic acid (pH = 1.3), and it is highly positively charged in strong acid conditions. DEXS multilayers were assembled from water solution (pH = 6.2), where the polysaccharide is fully negatively charged. Since the gel is negatively charged, the first PE layer deposited is a polycation (PLL or CHIT). The mixture was kept under constant stirring for 4−6 h. After each deposition, the excess of PE and buffer was removed by several ultracentrifugation cycles followed at each step by decantation and redispersion in water by vigorous shaking over at least 4 h. This sequence was repeated until the desired PE layers were deposited (see Scheme 3).

A similar procedure can be employed to prepare coated DEX-HEMA MGs [6]. A MG aqueous dispersion was mixed with the PE solution, and the PE was allowed to adsorb for 10 min under continuous shaking. The dispersion was then centrifuged, the supernatant removed and the MG redispersed in water to remove the non-adsorbed PE. As the MG is positively charged, the first layer is always a polyanion (pASP, PGA or DEXS). CHIT, PLL, HA, ALG were not suitable polycations since they caused strong MG aggregation. The only biopolycation that coated the DEX-HEMA microgels without aggregation was (pARG), a strong PE. Typically, four biopolymer bilayers were deposited.

The assembly of polypeptides onto PAA MGs was performed in solutions of high pH (>10) and low salt concentration [9,10]. After peptide binding was completed, the MGs were flushed with a solution of high electrolyte concentration (150 or 220 mM), thereby inducing PE detachment from the MGs due to reduced electrostatic attraction. This detachment was monitored through subsequent microgel reswelling in response to peptide release. To establish the final degree of peptide release, the MGs were equilibrated in the control solution again, as the MG volume was influenced by high electrolyte concentrations.

The deposition of PLL onto thiolated HA microgels was carried out at pH 6.5 by incubating the MGs in the PE solution for 10 min followed by centrifugation and three washing steps with NaCl solution [28]. After the desired number of HA/PLL layers was deposited, LbL-coated HA microgels were centrifuged and incubated in dithiothreitol (DTT) solution for 12 h at pH 7.0 with continuous shaking to remove the core of the MG. The hollow microcapsules were then washed with NaCl solution several times and incubated in EDC solution for 6 h to obtain shell crosslinked microcapsules. A very similar procedure was used to assemble PLL onto VB and hydrazide-modified HA, but in this case the pH was set at 7.4 [29,30].

## 5. Characterization Techniques

### 5.1. Electrophoretic Measurements

The zeta potential (ζ) is a term describing the electrokinetic potential in colloidal systems and is typically used to compute the surface charge on hard and rigid particles that arises from the potential difference between the immobile fluid layer bound to the dispersed particle and the medium. A high ζ value is related to the colloidal stability and is needed to prevent the suspension becoming unstable and aggregating. This is particularly relevant when the addition of salt is added which can have a destabilising effect on the dispersions. Generally a value above 25 mV (+ve or −ve) is needed to keep the system in a dispersed state. However, ζ is a parameter that cannot be calculated directly, but rather using a series of other models and the experimentally derived electrophoretic mobility μ_e_ (Equation (1)) [31]. At the turn of the 20^th^ century Smoluchowski utilizing the values of electric field strength, *E*, velocity of a dispersed particle, υ, dielectric constant of the dispersion medium, ε_r_, permittivity of free space (C²·N^−1^·m^−2^), ε_0_, and the dynamic viscosity of the dispersion medium (Pa s), η, derived an equation for ζ that can be applied to dispersed particles of any shape or concentration in most aqueous systems (Equation (2)) [32]:

(1) $${\text{μ}}_{\text{e}}\text{=}\frac{\text{υ}}{\text{E}}$$

(2) $${\text{μ}}_{\text{e}}=\frac{{\text{ε}}_{\text{r}}{\text{ε}}_{0}\text{ζ}}{\text{η}}$$

However, the limiting factor with this equation is when it is applied to nanocolloids in very low ionic strength solutions (near to pure water). As gels are not “hard-sphere” constructs,* i.e.*, typically porous and yielding, the electrophoretic mobility is not converted to the zeta potential, but instead used to calculate its surface charge density. Therefore, during the LbL assembly of PEs the system can be followed as a function of temperature by recording the electrophoretic mobility μ_e_ of the dilute aqueous mixtures after each deposition step.

### 5.2. Dynamic Light Scattering

Dynamic light scattering (DLS) is a commonly used non-invasive technique for the determination of particle sizes of proteins, colloids, emulsions, micelles, polymers and nanoparticles,* etc.* that can extend to around 1 nm. It works on the principle that when the solution is illuminated using a laser source, a time-dependent fluctuation takes place in the particles, altering their scattering intensity and therefore information on the time scale of the movement can be derived. This information can be obtained from the second order autocorrelation curve, which can be written as:

(3) $$g^{2}\text{(q;τ)=}\frac{<I\left(t\right)I\left(t+\text{τ}\right)>}{<I\left(t\right){>}^{2}}$$

*q* being the wave vector, τ the delay time and *I* the light intensity. The simplest approximation is to consider the first order autocorrelation function as a single exponential decay:

(4) $$g^{2}\text{(q;τ)=exp(-Γτ)}$$

where Γ is the decay rate. This is correct for a monodisperse population. The translational diffusion coefficient *D_t_* can be determined at a certain angle taking into account the expressions:

(5) $$\text{Γ}=q^{2}D_{t}$$

(6) $$\text{q=}\frac{4\text{π}n_{0}}{\text{λ}}\text{sin}\left(\frac{\text{θ}}{2}\right)$$

where λ is the incident laser wavelength, *n*_0_ the sample refractive index and θ the angle at which the detector is located with respect to the sample cell. Generally, a cumulant analysis is applied to estimate the particle size [33]. Using this approach, information about the variance of the system can be obtained as:

(7) $$g^{1}\text{(q,τ)=exp(}-\bar{\text{Γ}}\text{τ)}\left(1+\frac{{\text{μ}}_{2}}{2!}{\text{τ}}^{2}-\frac{{\text{μ}}_{3}}{3!}{\text{τ}}^{3}+\cdots \cdots \right)$$

$\bar{\text{Γ}}$ being the average decay rate and ${\text{μ}}_{2}{\bar{\text{Γ}}}^{-2}$ the second order polydispersity index (or an indication of the variance). Similarly, the *z*-averaged translational diffusion coefficient *D_z_* can be calculated at a single angle by the equation:

(8) $$\bar{\text{Γ}}=q^{2}D_{Z}$$

The Stokes-Einstein equation can then be applied to calculate the hydrodynamic radius *R*_h_ from the diffusion coefficient [34]:

(9) $$D_{Z}=\frac{K_{\text{B}}T}{6\text{πη}R_{\text{h}}}$$

DLS measurements are generally carried out on highly diluted gel solutions after each deposition stage* vs.* temperature (*i.e.*, heating from 20 to 40 °C and cooling down to 20 °C, in steps of 2 °C to obtain a complete cycle).

### 5.3. Environmental Scanning Electron Microscopy (ESEM)

Environmental scanning electron microscopy (ESEM) can be used to examine the morphological characteristics of both uncoated and LbL-coated ensembles in the hydrated state, avoiding significant structural alterations that might occur when drying the porous gels. ESEM of these types of samples typically occurs under the following conditions: water vapour pressure (0.5–0.6 kPa), room temperature and for only short periods of time (approximately 3 min).

### 5.4. Attenuated Total Reflectance FTIR Spectroscopy

Attenuated total reflectance-Fourier transform infrared spectroscopy (ATR-FTIR) is used to monitor the interaction between the gels and the PEs non-destructively. Depending on the PEs assembly and consequently its interaction with the gel, it can cause shifts in peak positions, intensity and the shape of the FTIR bands. Key differences can normally be identified within the fingerprint region of the spectra, with detailed analysis including peak deconvolution.

### 5.5. Confocal Microscopy and Fluorescence Correlation Spectroscopy

Confocal microscopy can be used to visualize the LbL assembly on thermoresponsive MGs. Combining this approach with laser scanning and fluorescent labelling results in the technique known as Confocal laser scanning microscopy (CSLM). The end result is a 3D high resolution image of the material. However, for NGs, Fluorescence Correlation Spectroscopy (FCS) is needed to be able to get any quantitative information regarding the LbL deposition process using auto- and cross-correlation functions of the fluorescently labelled PEs [35]. FCS was initially used to detect the diffusion coefficient of nanomolar concentrations of fluorescently labelled species in which spontaneous fluorescence intensity fluctuations are monitored as the species enter and leave the limit of detection (called autocorrelation function, ACF). Typically, FCS is employed in confocal microscopy or two-photon excitation microscopy, where the laser beam is focused on the sample and correlation analysis of temporal fluctuations of the fluorescence intensity (due to diffusion, physical or chemical reactions, aggregation,* etc.*) are measured using the ACF. Calibration before measurement is needed for quantitative use, with information on the size and shape of the excitation/detection volume required, all dependent on laser power and the sample’s optical saturation and refractive index. To bypass these limitations the use of two overlapping excitation foci at a precisely known distance (dual focus Fluorescence Correlation Spectroscopy (2*f*-FCS)) can be used, thus creating an external length scale with a similar accuracy to the well-known DLS or small angle neutron scattering (SANS) as proposed by Dertinger* et al.* [36]. 2*f*-FCS data assessment can be regulated in order to interpret finite size effects in the colloidal and macromolecular systems. Advancements in this technique have seen the use of heating cells inserted within inverted microscope setups and the use of two-colour fluorescence cross-correlation spectroscopy. The latter enables the PEs that can merge with the PEs of other layers or adsorbed within the gel to be identified. This is possible via cross-correlating two or more fluorescent channels, permitting the fluorescent free labelled PE and those bound to the NG for example to be distinguished due to diffusion differences, and hence, making visualization of the layer binding possible.

### 5.6. Atomic Force Microscopy

Atomic force microscopy (AFM) allows very high resolution 3D images of the morphological properties of samples to be analysed by scanning their surface using a cantilever and probe. The materials to be analysed are typically deposited onto a mica substrate, dried and tested using a tapping mode. In this function the cantilever oscillates at its resonant frequency at room temperature in air, and as it is brought to the sample surface it is caused to deflect according to Hooke’s law, with the deflection normally measured by a “*beam bounce*” method, with the computed deflections used to generate a surface topography map. The nanomechanical properties of thin films such as LbL constructions can also be tested using AFM by changing the cantilever probe to a sphere tip and applying it as a type of microindenter [29].

### 5.7. Swelling Experiments

Two main techniques are employed for measuring the swell ratio of micro/nano-gels. One is a gravimetric approach in which the sample is initially weighed, subsequently immersed in a solvent at the desired temperature for 48 h and weighed again. The swell ratio is determined from the weights and the ratio of the densities of the solvent to the gel. Although this is a simple and low cost method, it is not accurate when using volatile solvents; further, it is difficult to determine when the steady state is reached. The other technique consists of the use of a probe to measure the change in the gel height as its swells. Assuming that the gel swells isotropically, the swell ratio can be calculated from the change in height. This allows the determinination of both the temporary and the steady state swell ratio.

### 5.8. Tensile and Compression Tests

The stiffness and strength of LbL films can be estimated by tensile or compression tests performed using an Instron mechanical tester. Typically, gels are allowed to swell at the desired temperature, wiped lightly, and then stressed or compressed at a typical rate between 0.2 and 5.0 mm/min until failure. To determine the tensile properties, samples are secured in custom grips with sandpaper and tested in uniaxial tension. In compression, the sample is placed between two plates and the pressure is applied to its surface. The Young’s or compression modulus is calculated as the slope of the linear region of the stress-strain curve and the ultimate strain is obtained as the strain at failure. At least three samples should be tested to get an average value.

## 6. Characterization of Biopolymer-Coated Gels

### 6.1. Morphological Characterization

**Figure 1:**
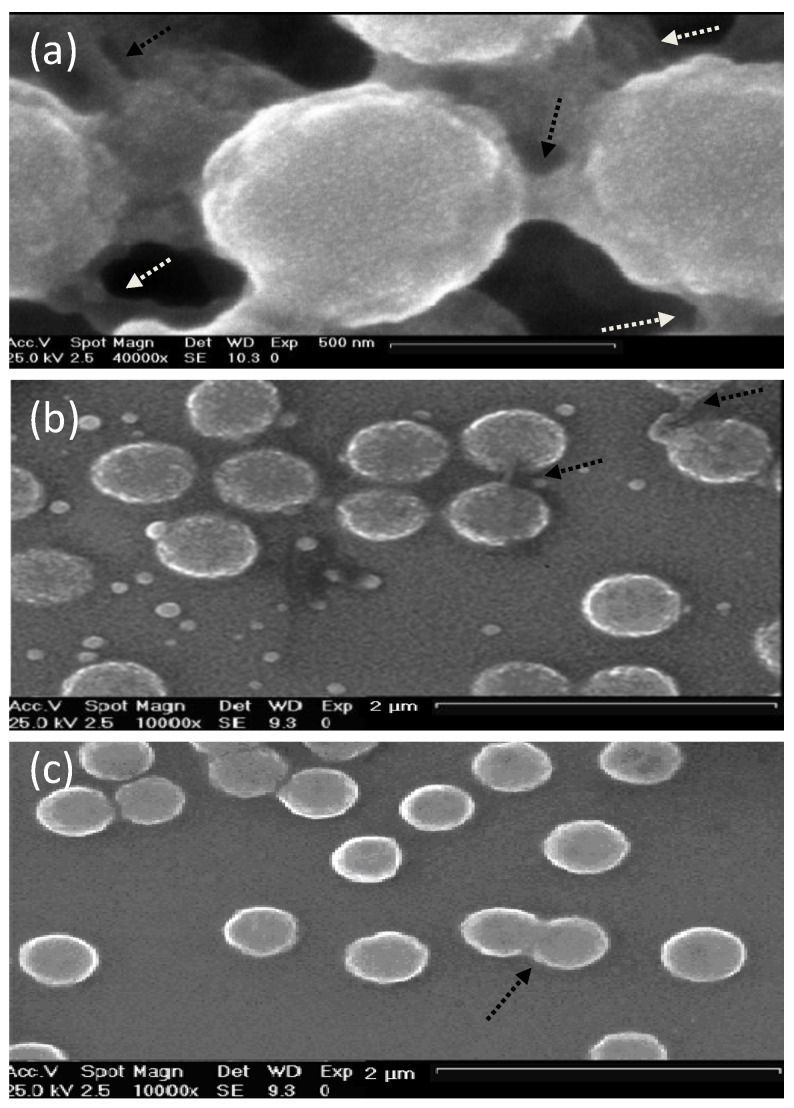
Typical environmental scanning electron microscopy (ESEM) micrographs of: (**a**) bare NG; (**b**) NG/(PLL/PGA) and (**c**) NG/(CHIT/dextran sulfate (DEXS)) in the hydrated state. Reproduced with permission from [8], copyright 2010, Elsevier.

The morphology of uncoated and coated-MGs (or NGs) in the hydrated state can be observed directly by ESEM. Diez-Pascual and Wong [8] analysed the morphology of PNIPAM-*co*-MAA coated with polypeptides and polysaccharides, as illustrated in Figure 1. The image of the pure NG (Figure 1a) showed soft, rough and porous nanospheres interconnected by bridges. In contrast, the PLL- and CHIT-coated NGs (Figure 1b,c, respectively) were more separated and presented more individual particles with few connections; analogous morphology has been described for all coated NGs [8]. The comparison of NG/PLL and NG/CHIT images revealed that polypeptide-coated systems partially maintain the rough surface of the uncoated NG, while those covered with polysaccharides exhibit a smoother structure. This behaviour could be related to differences in layer thickness, as reported for biopolymers assembled onto flat substrates [37]. Polypeptides have more propensity to rearrange and form complexes with both PLL and PGA in β sheet antiparallel conformation, with extensive interpenetration and strong ionic interaction between biopolymers from adjacent layers, which lead to a highly compact structure that fits easily onto the NG surface, thus retaining its rough morphology. This is consistent with the images reported by De Geest* et al.* [6] for pARG-coated DEX-HEMA MGs showing very rough microspheres attached to each other.

Though, as can be seen in Figure 1 the PNIPAM-*co*-MAA systems presented small evenly sized spherical particles, the average diameter of which, for the pure NG, NG/CHIT and NG/PLL was 692, 462 and 590 nm, and those of the bi-coated samples, NG/(CHIT/DEXS) and NG/(PLL/PGA) were 538 and 676 nm, respectively, in agreement with measurements obtained from DLS experiments in the swollen state [8]. The decrease in diameter upon the addition of the initial polycation layer and *vice versa* increasing with addition of the second polyanion coating is not untypical and has been seen for a number of systems below the VPTT temperature, such as PLL and PGA coated-PNIPAM-*co*-MAA MGs [27] as well as NGs coated with synthetic polymers such as PDACMAC or PPS [19]. The electrostatic attraction between the negatively charged NG and the positively charged polycation layer induces a contraction, whilst the addition of the next polyanion layer affectively shares the positive charge with the NG, weakening the interaction and results in a relaxation in the structure, and hence, increase in diameter. Overall, SEM images not only provide evidence of the success of the LbL assembly process, but also demonstrate that the morphology of the NGs can be tuned by surface adsorption of the PE layers.

**Figure 2:**
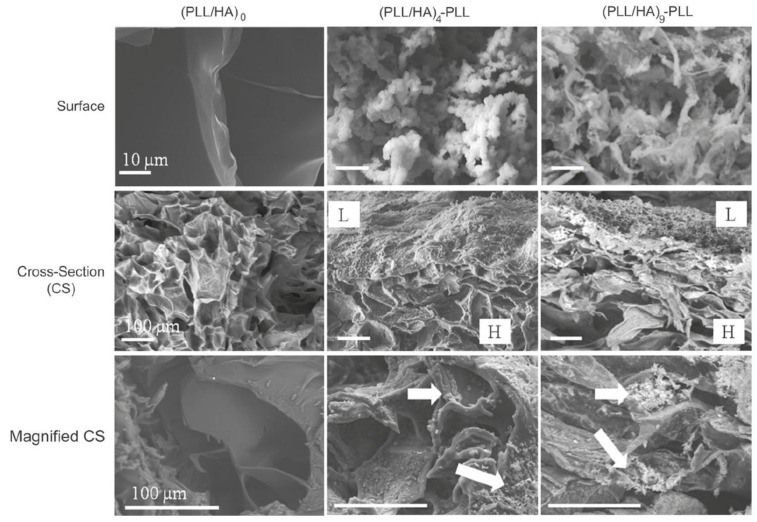
Scanning electron microscope (SEM) images of the top surface and cross-sections of HA MGs (left column) and microgels (MGs) coated with (PLL-HA)_4_-PLL, and (PLL-HA)_9_-PLL films (middle and right columns, respectively). The arrows indicate diffusion of the PEs into the gel. Reprinted with permission from [37], copyright 2011, Elsevier.

The morphology of HA MGs LbL coated with PLL/HA layers was investigated by Yamanlar *et al.* [37] (Figure 2). SEM images of the bare MG (left column) revealed a smooth flaky surface, in contrast to the morphology found for PNIPAM based gels [8,19,27]. However, systems with four and nine PLL/HA bilayers (middle and right columns, respectively), displayed a rough surface with granular or hair-like structures. The outer surface of the coated MGs showed formation of the LbL film (labelled as L in the micrographs), and the film thickness was higher for the assembly with (PLL/HA)_9_-PLL as compared to (PLL/HA)_4_-PLL. Moreover, granular features were visible in the bulk region of the hydrogel (marked as H) indicating diffusion of the PEs into the gel (indicated by arrows). When the HA MG was brought into contact with the PLL, some of the polycations diffused into the bulk gel, likely forming the initial phase of multilayer growth inside the intrinsic pores due to negatively charged HA. However, as the deposition steps increased, the layers were formed on the surface masking the MG pores as validated by the SEM images (top row).

**Figure 3:**
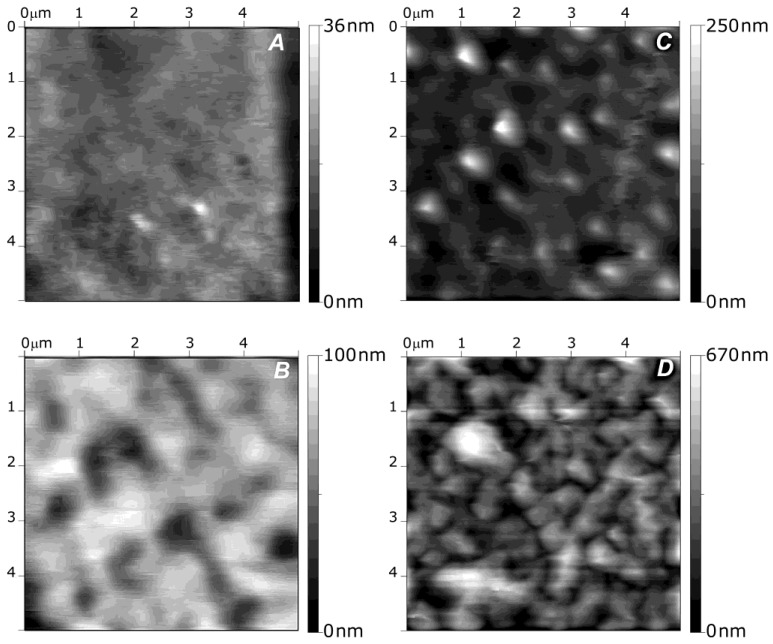
Atomic force microscopy (AFM) images of different alkylamino hydrazide-modified HA/PLL systems with 18 layer pairs: (**A**) linear C6 derivative with DS of 5%; (**B**) branched C10 derivative with DS of 5%; (**C**) linear C10 derivative with DS of 5% and (**D**) linear C10 derivative with DS of 10%. Reproduced with permission from [30], copyright 2009, American Chemical Society.

Kadi* et al.* [30] investigated the morphology of alkylamino hydrazide-modified HA derivatives. Figure 3 shows AFM images of the surface of the systems containing 18 modified HA/PLL layer pairs. Two characteristic morphologies were observed: those incorporating alkylated derivatives with six and eight carbon atoms showed a smooth surface, similar to that of neat HA/PLL, with a mean roughness of 4 nm (Figure 3a). However, films based on derivatives with 10 carbon atoms displayed a rough surface (Figure 3b–d), particularly that with a DS of 5% and linear alkyl chains (Figure 3d) which had an average roughness of 98.2 nm. On the other hand, “small grains” or microaggregates were visible at the surface of systems with 10 carbon atoms and linear chains. This presence of small aggregates whose size depends on the alkyl chain length and the DS is consistent with previous LbL films made of PAA and hydrophobically modified poly(ethylene oxide). Thus, it was proposed that derivatives with long chains and high DS had an irregular internal structure, with hydrophobic nanodomains coexisting with hydrophilic domains. Similar behaviour was reported for VB-g-HA/PLL, where the mean surface roughness of the systems increased from 0.3 to 9 nm when the grafting degree rose from 14% to 37% [29].

### 6.2. ATR-FTIR Spectra

**Figure 4:**
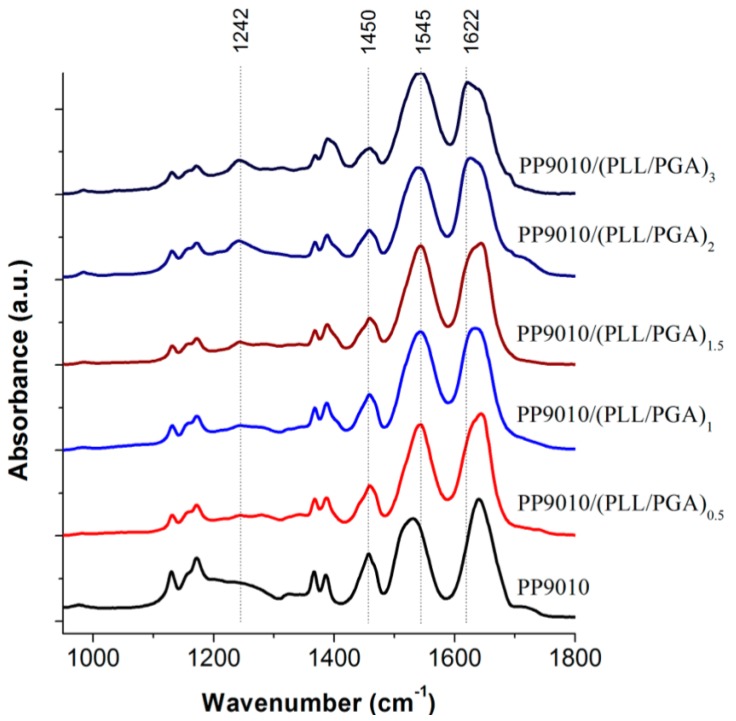
Attenuated total reflectance FT-IR spectroscopy (ATR-FTIR) spectra of polypeptide-coated P(NIPAM-*co*-MAA) MGs. Reproduced with permission from [27], copyright 2012, American Chemical Society.

ATR-FTIR spectroscopy was used by Hammond and co-workers [27] as shown in Figure 4 to analyse the interactions between biopolymers and P(NIPAM-*co*-MAA) MGs. Characterisation of materials is difficult due to the fact that the amide vibrations overlap with the characteristic PE bands. In addition, the polypeptide bands depend very much on their conformation (α-helix, β-sheet, or random coil), hydrogen bonding and vibrational interactions between the peptide groups. However, ATR-FTIR can be very informative, for example, in the case of the MG, it consists of two strong amide I and amide II bands at 1641 and 1531 cm^−^^1^, and a weak amide III band at 1242 cm^−1^ from the PNIPAM segments. Adsorption of the polypeptides leads to an intensity increase in the amide II band, and a decrease in the shoulder band at 1707 cm^−^^1^, which corresponds to the C=O stretching of the unprotonated carboxylic groups of PMAA, suggesting that the PE layering process occurs primarily through ionic interactions.

Deposition of PLL on PNIPAM led to the shift in the amide bands to much higher energy states, characteristic of the vibrations from cross-linked homopolymers (δ_NH_ at 1543 cm^−^^1^, intramolecular H-bonded ν_C=O_ at 1644 cm^−^^1^ and free ν_C=O_ at 1669 cm^−^^1^). Again, the consequent adsorption of PGA altered intramolecular H-bonded ν_C=O_ (1632 cm^−^^1^) and free ν_C=O_ (1653 cm^−^^1^) to lower wavenumbers similar to that of the native NG, whereas the intensity of the free ν_C=O_ of PMAA COOH group was conserved, suggesting that the PNIPAM segments are strongly involved in H-bonding. Additionally, in the higher wavenumber region the contribution of PLL ν_N__–__H_ and PGA ν_O__–__H_ led to an increase in intensity and broadening of the N–H stretching between 3000 and 3450 cm^−^^1^ relative to the isopropyl and alkyl vibrations (2850–3000 cm^−^^1^). Subsequent PE assembly layering did not significantly alter the spectra, strengthening the previous discussion.

### 6.3. Surface Charge Density

**Figure 5:**
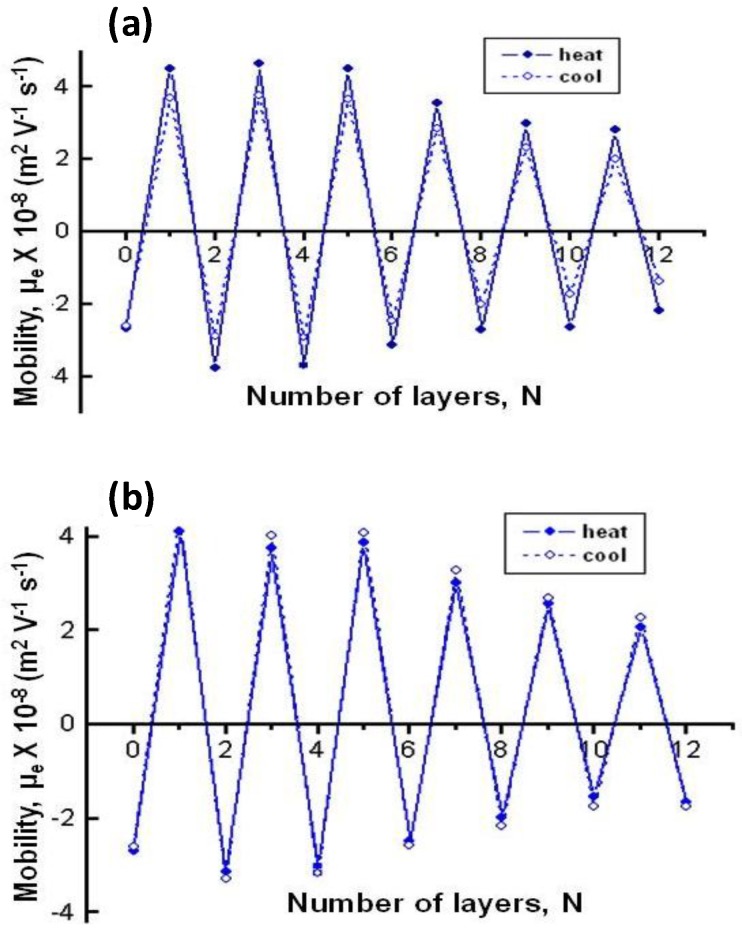
Electrophoretic mobility μ_e_ at 20 °C* vs.* number of layers deposited on PNiPAM-*co*-MAA NGs: (**a**) NG/(PLL/PGA) and (**b**) NG/(CHIT/DEXS). Reproduced with permission from [8], copyright 2010, Elsevier.

Electrophoretic mobility (μ_e_) measurements can be employed to monitor the subsequent deposition of PEs onto gels after each adsorption step. µ_e_ values at 20 °C of PNIPAM-*co*-MAA NGs coated with polypeptides and polysaccharides as a function of the number of layers deposited are compared in Figure 5, with an analogous trend found for other temperatures tested [8]. µ_e_ of the bare PNIPAM-*co*-MAA NG in aqueous solution is −2.7 × 10^−8^ m^2^ V^−1^ s^−1^ (negatively charged), due to the deprotonation of most of the acid groups of MAA (pH = 4.4). After adsorption of each polycation and polyanion layer, the surface charge becomes positive and negative, respectively, consistent with the behaviour observed by other authors dealing with similar biopolyelectrolytes and different substrates [38,39]. Nevertheless, the surface charge is not completely compensated in terms of absolute values, in contrast to what is observed for LbL assembly on flat surfaces or rigid particles. This suggests that interactions between the adsorbed PE films and the NG take place, and might be related to incomplete coverage of the NG surface and/or interpenetration with the PEs. A similar trend of charge reversal after each PE deposition was found for PLL/PGA-coated PNIPAM-*co*-MAA MGs [27], and confirms the possibility of the LbL assembly of biopolyelectrolytes onto thermoresponsive gels. The values of µ_e_ obtained are quite high, consistent with those found by Haidar* et al.* [40] for liposomes coated by biopolymers, and demonstrates the high stability of these systems. Figure 5 reveals that µ_e_ behaviour is similar for both types of PEs, although the larger charge reversal (+4.3 × 10^−8^ m^2^ V^−1^ s^−1^) is obtained after deposition of the first CHIT layer compared to PLL (+3.9 × 10^−8^ m^2^ V^−1^ s^−1^). This could be understood by considering that CHIT has more functional groups and higher charge density since it is assembled from solutions with low pH and ionic strength, and tries to adopt an extended rod-like configuration, leading to a larger excess of positive charges onto the NG surface. Interestingly, on assembly of the first polyanion layer, the absolute *µ_e_* value is higher for DEXS-terminated NG compared to PGA-terminated NG, likely due to their dissimilar charge density, provoked by the different assembly conditions. As indicated in the introduction, when these weak PEs are incorporated into the multilayer system, their degree of ionization can vary considerably from the solution value owing to the influence of the local environment via electrostatic and hydrophobic effects. Therefore, when DEXS is adsorbed, the presence of many oppositely charged cationic groups will augment the degree of ionization of this polyanion compared to the solution value, resulting in a high surface charge density. However, when PGA is adsorbed, there are less cationic groups on the surface, because some of the amine groups of PLL are compensated by salt counterions, consequently reducing the magnitude of the electrostatic effects. Furthermore, the apparent pK_a_ of PGA can be increased and that of PLL reduced due to hydrophobic effects, which would diminish the degree of ionization of these PEs in the multilayer. Thus, the pK_a_ of the carboxylic groups of PGA has been reported to fall slightly upon incorporation in the multilayer [41], though it increases up to ~4.5 in the presence of salt. Similarly, a pK_a_ drop from ~10.5 in solution to 9.8 was detected for the amine groups of PLL, interpreted in terms of a proton release from the NH_3_^+^ due to the interaction with the negatively charged groups of PGA. All the above mentioned changes in the degree of ionization of the different groups should have an effect on the charge balance during the LbL assembly.

Figure 5 also shows a systematic drop in µ_e_ with increasing number of layers deposited onto the NG, regardless of the type of PE. This is a key difference with the LbL assembly on hard and rigid particles [38], where the magnitude of charge reversal does not change. LbL films can be considered as ordered non-equilibrium arrangements in which the PE segments have a lot of freedom for mobility [42]. The soft and porous nature of the gels allows penetration of the PEs to a certain extent and mutual interactions that are not feasible with solid and rigid substrates. PEs can diffuse in and out, thereby neutralizing charges. Rupture and formation of ion pairs can occur not only between polycations and polyanions but also between polycations and gels; all these facts account for the fall in µ_e_ with increasing number of layers. This behaviour indicates that the electrostatic interactions and the intrinsic mechanism of charge compensation primarily contribute to the multilayer formation during the adsorption of the initial layers, whereas the secondary interactions become the driving force of the assembly with increasing number of deposition stages, which is reflected in a reduction in µ_e_. However, a different trend was reported for HA/PLL multilayer films, where the magnitude of the zeta potential (ζ) even slightly increased with the deposition steps, likely because HA acts both as template and polyanion in this system [28].

**Figure 6:**
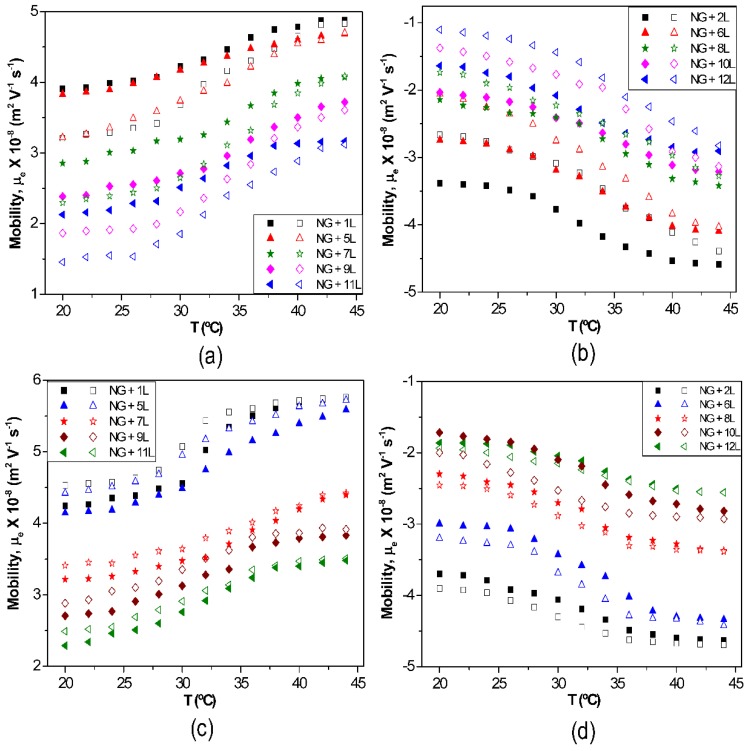
Electrophoretic mobility μ_e_ *vs.* temperature for different biopolymer-terminated PNIPAM-*co*-MAA: (**a**) PLL; (**b**) PGA; (**c**) CHIT and (**d**) DEXS. Reproduced with permission from [8], copyright 2010, Elsevier.

The effect of temperature on µ_e_ for biopolymer-coated PNIPAM-*co*-MAA NGs is shown in Figure 6: (a) PLL, (b) PGA, (c) CHIT and (d) DEXS [8]. For all the systems, the absolute value of µ_e_ rises with increasing temperature owing to the inherent thermoresponsivity of the NG. This is explained by considering that the gel swelling is entropically driven and depends on the formation of hydrogen bonds between the amide groups of PNIPAM and the water molecules. The rise in temperature breaks these stabilizing H-bonds, hence the NG shrinks, pushing the charges out, and provoking an increase in the surface charge density. Since the NG has water inside, in the collapsed state, on cooling some trapped segments are able to reorganize as water flows into the gel, and this could account for the hysteresis found in µ_e_ between the heating and the cooling cycles. As can be observed from Figure 6, the aforementioned hysteresis is more significant for NGs coated with polypeptides compared to those coated with polysaccharides, indicating a higher extent of internal rearrangement in the former biopolymers. This behaviour could also be ascribed to the high ionic strength of the PLL and PGA dipping solutions. As indicated previously, salt counter ions occluded within the multilayer, swell the ensemble by screening the electrostatic forces that join the layers, thereby provoking higher mobility of the polymer chains, hence restructuration of the entire ensemble, leading to a noticeable hysteresis. However, in the case of high *M*_w_ polysaccharides assembled from water solutions, less rearranging is allowed, albeit they also undergo small conformational changes between different helical structures caused by the swelling and collapsing of the NG. For the same number of layers and temperature, polysaccharides display higher µ_e_ values, attributed to their larger number of functional groups and charges, and try to adopt a stretched conformation, they thus have more charges at the NG surface, while polypeptides adopt a more coiled configuration, exposing fewer charges on the surface.

The sequential LbL deposition onto MGs has also been monitored by contact angle measurements. For instance, Yamanlar* et al.* [37] studied the multilayer growth of PLL and HA onto HA MGs. The contact angle of uncoated HA was 36.5°, and increased to 44° upon deposition of the first PLL layer. Subsequent deposition of each HA and PLL layers led to similar results (*i.e.*, low and high contact angles for HA and PLL, respectively) indicating the multilayer buildup on the outer surface of the MG. Therefore, the polycationic PLL layers were more hydrophobic than the polyanionic HA ones. Similarly, Kolasinska* et al.* [43] reported that PLL terminated multilayers were more hydrophobic than PGA terminated ones. The authors suggested that the electric charge density is lower when the polycation forms the outermost layer. Another proposed reason was the more favourable orientation of water molecules at the negatively charged surfaces. For the first three layer pairs, there was little change in the contact angle values. However, when the number of the layers grew, the contact angle in each deposition increased, ascribed to the interlayer mixing during PLL/HA film growth, since the polypeptide can diffuse in and out of the layers.

### 6.4. Hydrodynamic Size

**Figure 7:**
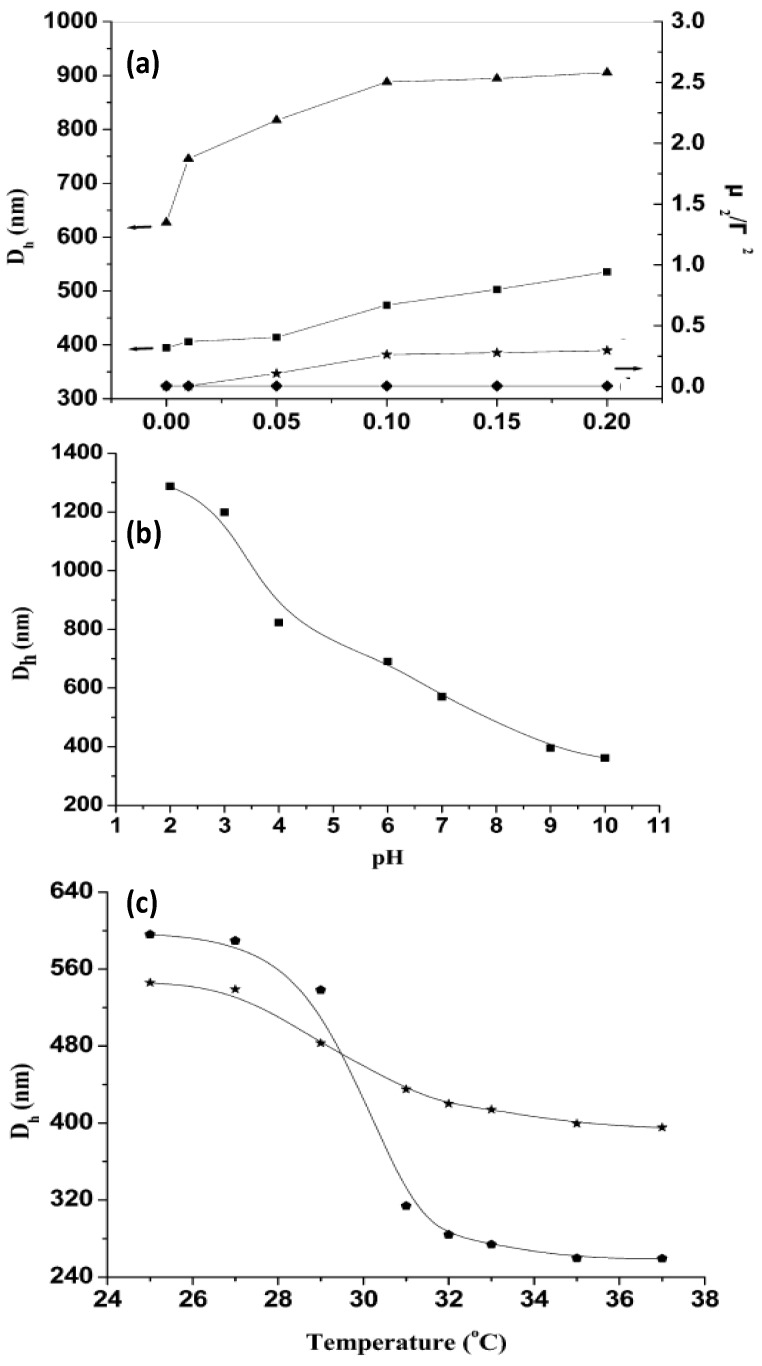
(**a**) Influence of ionic strength on the hydrodynamic diameter *D*_h_ and polydispersity index (μ_2_/Г^2^) of PNIPAM-grafted (CHIT/ALG)_4_ hollow microspheres (▲ and ♦) and (CHIT/ALG)_4_ hollow microspheres (■ and ★); (**b**) pH dependence of *D*_h_ of PNIPAM-grafted (CHIT/ALG)_4_ hollow microspheres at 25 °C; (**c**) Temperature dependence of *D*_h_ of PNIPAM-grafted (CHIT/ALG)_4_ microspheres with low (★) and high (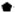) grafting degree. Reproduced with permission from [44], copyright 2012, American Chemical Society.

The thermoresponsive behaviour of uncoated and coated gels can be monitored by DLS. Mu* et al.* [44] investigated the influence of the ionic strength, pH and temperature on the hydrodynamic diameter (*D*_h_) of PNIPAM-grafted (CHIT/ALG)_4_ hollow microspheres (Figure 7). For comparison, the size of (CHIT/ALG)_4_ microspheres was also investigated.

It was found that the increase in the ionic strength from 0 to 0.20 mol/L NaCl increased *D*_h_ of the polysaccharides multilayers from ~400 to 540 nm, whilst that of PNIPAM-grafted (CHIT/ALG)_4_ increased from ~630 to 910 nm (Figure 7a). This behaviour was explained by considering that the presence of salt weakens the electrostatic interaction between ALG and CHIT, hence the PE chains are stretched and the size of the hollow microspheres rises. Moreover, the polydispersity index (μ_2_/Γ^2^) of the microspheres composed only of polysaccharides increased steadily with increasing ionic strength, indicative of aggregation during the measurements, whilst that of the assemblies with PNIPAM remained almost constant, demonstrating that the grafted PNIPAM prevents flocculation among the microspheres in solutions with higher salt concentration.

On the other hand, *D*_h_ of PNIPAM-grafted (CHIT/ALG)_4_ decreased from ~1290 to 360 nm with increasing pH (Figure 7b). At low pH (<4), CHIT was hardly ionized, hence was scarcely crosslinked with ALG, leading to a big PE shell. Between pH 4 and 7, there is a strong electrostatic attraction between both PEs, resulting in a reduction in *D*_h_. The ionization of the amine groups of CHIT decreased greatly when the solution pH increased above 6.0 (close to the pK_a_ of CHIT) and at pH > 7.5 less than 10% of its amine groups were ionized, provoking the shrinkage of the polysaccharide chains, and hence a remarkable reduction in size. The results demonstrate that the PE shells of the hollow microspheres were pH-sensitive.

The influence of temperature on *D*_h_ was also analysed (Figure 7c). Clearly, the VPTT occurs at around 32 °C. Upon heating from 25 to 37 °C, *D*_h_ of the grafted ensemble with lower degree of grafting decreased from 545 to 400 nm, whilst that of the system with higher grafting degree dropped from 600 to 260 nm, corroborating that the higher PNIPAM content, the larger the thermosensitivity of the system. Overall, the hollow microspheres retained the pH, ionic strength and thermosensitivity, and they did not disintegrate with variations in these parameters.

**Figure 8:**
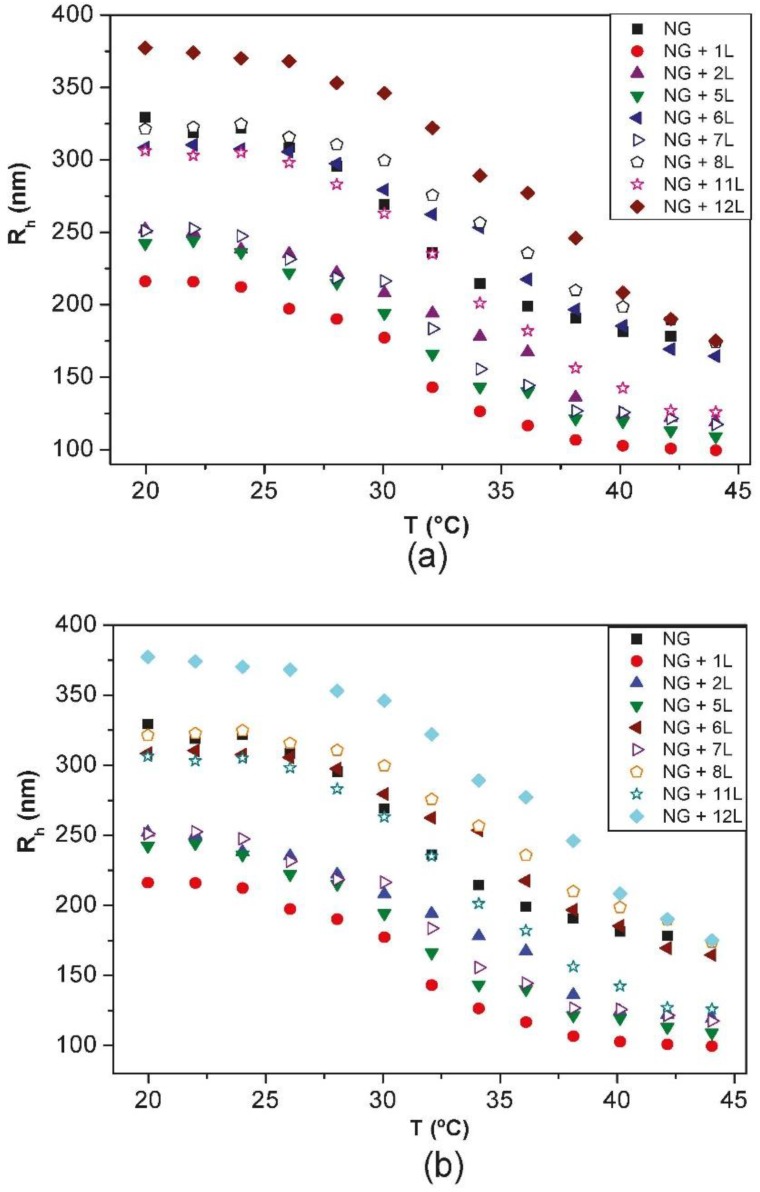
Hydrodynamic radius *R*_h_* vs.* temperature for PNIPAM-*co*-MAA with different layers of: (**a**) PLL/PGA and (**b**) CHIT/DEXS. For clarity, only the heating cycles are shown. Reproduced with permission from [8], copyright 2010, Elsevier.

Diez-Pascual and Wong [8] compared the change in the hydrodynamic radius *R*_h_ with increasing temperature for PNIPAM-*co*-MAA NGs coated with different layers of PLL/PGA and CHIT/DEXS (Figure 8). The bare NG in water (pH = 4.4) possesses a *R*_h_ of 330 nm at 20 °C. During heating, it collapses to ~180 nm, and on cooling it recovers the original size. Upon adsorption of the first polycation layer there is a strong decrease in the NG size, albeit the coated NGs maintain the thermoresponsive behaviour, and on heating they still undergo the transition to a collapsed state. The deposition of the second layer provokes an increase in size, and the temperature sensitivity of the coated NG is also retained. This “odd-even” effect was corroborated by ESEM images. The size of one-layer polysaccharide coated NG in the collapsed state is ~100 nm, whereas PLL-coated NG shrinks to ~170 nm. To understand the differences between the behaviour of NGs coated with polypeptides and polysaccharides, the PE charge density and secondary cooperative interactions that contribute to the film formation have to be considered. The polysaccharide displays a larger number of ionized functional groups that can shield the negative charges of the NG, causing a rearrangement of the gel in order to adopt a more coiled and compact conformation, resulting in a smaller *R_h_*. Further, upon deposition of CHIT, strong H-bonds can be formed between its hydroxyl groups and the solvent at the expense of breaking stabilizing interactions between the amide groups of PNIPAM and the water molecules, resulting in a decrease in size. Upon approach of the DEXS layer, the H-bonds of the confined CHIT are shared between the solvent and the polyanion; this weakens the interactions with the solvent, leaving some water molecules mobile again and able to penetrate the NG, causing the assembly to expand. This explains how PNIPAM-*co*-MAA/(CHIT/DEXS) presents the mentioned “odd-even” effect in both swollen and collapsed states.

On the other hand, polypeptides are assembled from neutral solutions with high ionic strength, where the charges of both the NG and the PEs are screened. Consequently, the NG-PLL electrostatic attraction is weaker than the NG-CHIT one, hence the decrease in size is less pronounced. Furthermore, secondary cooperative interactions play a key role in the construction process. After adsorption of the PGA layer, both biopolymers also interact via H-bonds and hydrophobic interactions. To attain the electrical neutrality necessary for the multilayer buildup, part of the polyanion charge is compensated by salt counterions that get blocked within the film structure. During the washing stages, the osmotic pressure acts as the driving force for the swelling of the system. The change in ionic strength leads to a difference in the chemical potential between the ions inside and outside the coated NGs, and consequently an exchange process of ionic pairs takes place between the multilayer and the solution until the chemical potential is equilibrated, and the overall result is a rise in *R*_h_.

**Figure 9:**
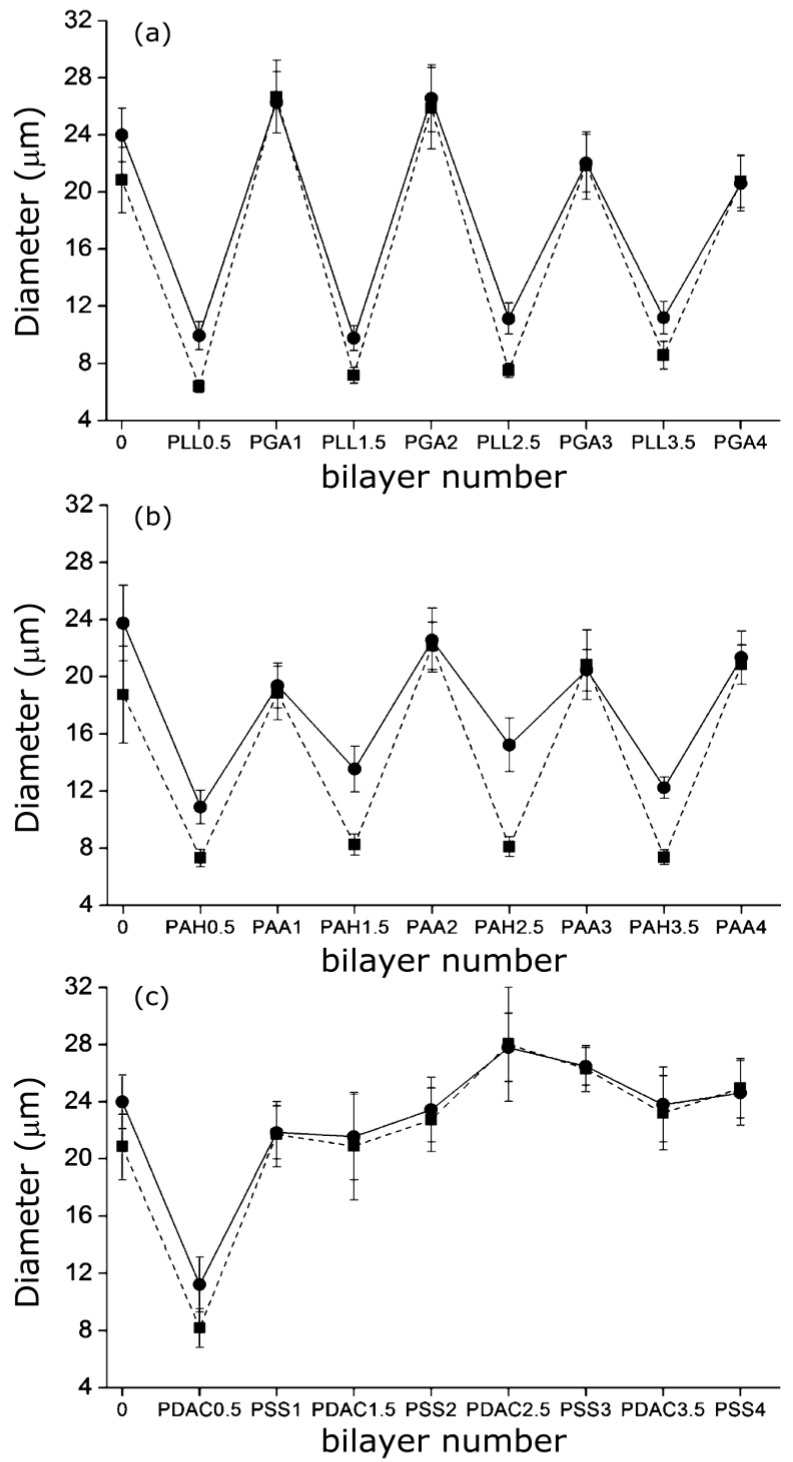
Hydrodynamic diameter of PNIPAM-*co*-MAA MGs upon LbL assembly of: (**a**) PLL/PGA; (**b**) PAH/PAA and (**c**) PDACMAC/PSS at 24 °C (circles) and 37 °C (squares). Reproduced with permission from [27], copyright 2012, American Chemical Society.

A similar “odd-even” effect both in the swollen and collapsed states was reported by Costa* et al.* [27] for PNIPAM-*co*-MAA MGs. They compared the hydrodynamic diameter* vs.* number of layers for MGs coated with PLL/PGA, PDACMAC/PSS and PAH/PAA (Figure 9), and found that the size and thermoresponsive behaviour was significantly influenced by the outermost layer.

Assemblies with polypeptides showed the stronger changes in *D*_h_ and systematically retained their thermoresponsive behaviour (Figure 9a), ascribed to the PE inter-diffusion during film buildup, hence the polycation/polyanion effect on the gel behaviour was easily reversed. In contrast, assemblies with strong PEs only changed the MG responsiveness upon addition of the first two layers (Figure 9c), attributable to the strength of their ionic complexes and the lower mobility of the PE chains that impair any effect of further layers on the overall responsiveness of the LbL assembled microgel. The swelling behaviour of coated gels can be characterized through the swelling ratio defined as (*D*_hswollen_/*D*_hcollapse_)^3^. In the case of PNIPAM-*co*-MAA MGs, this ratio is higher for biopolymer coated systems compared to those coated by synthetic polymers.

**Figure 10:**
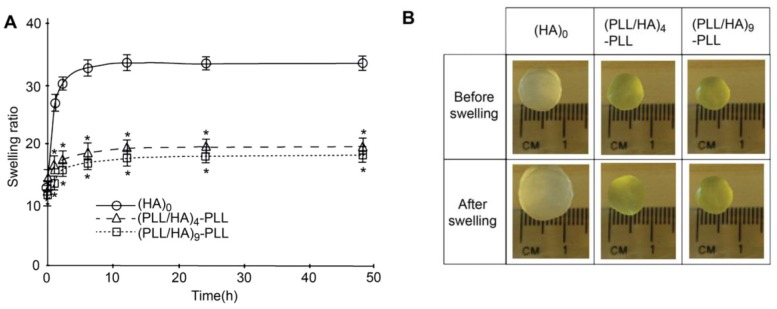
(**A**) Swelling ratio of bare HA MGs, (PLL/HA)_4_-PLL and (PLL/HA)_9_-PLL and (**B**) Photographs of the uncoated and coated microgels before and after immersion in phosphate buffered saline. Reproduced with permission from [37], copyright 2011, Elsevier.

Other authors have carried out swelling studies on HA MGs coated with PLL/HA multilayers [37]. The samples were immersed in phosphate buffered saline for predetermined times, quickly wiped to remove the residual liquid and their swollen weight recorded. Subsequently, they were dried in a vacuum oven and weighed again to determine their dry weight. It was found that coated gels displayed a lower degree of swelling compared to the unadulterated MG (Figure 10a), explainable in terms of PE diffusion inside the pores as well as increased hydrophobicity of the surfaces. According to SEM images (Figure 2), interpenetration of the PEs inside the pores occurs during multilayer formation, causing their partial or complete blockage, likely affecting the swelling properties of the coated gels. Further, as mentioned earlier, contact angle measurements suggest that PLL increased the surface hydrophobicity. Thus, the increased hydrophobicity and reduced permeability should result in lower swelling for the coated MGs. Indeed, when hydrophobic forces dominate, the tendency to diminish the polymer/water interface leads to reduced swelling. On the other hand, there was hardly any effect of the number of PE layers on the swelling ratio, as can be deduced from the comparison of the photographs of (PLL/HA)_4_-PLL and (PLL/HA)_9_-PLL before and after immersion in the buffer (Figure 10b).

### 6.5. Bilayer Thickness

**Figure 11:**
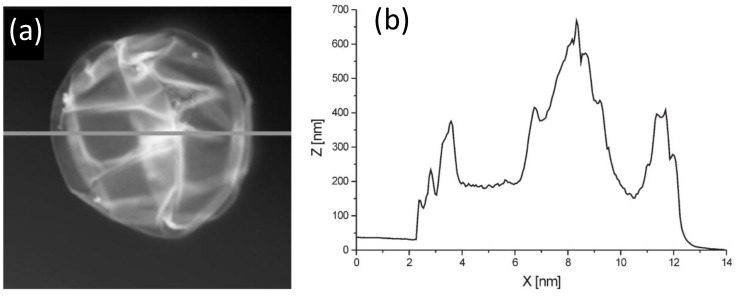
(**a**) AFM image of a hollow DEX-HEMA MG coated with four (DEXS/pARG) bilayers and (**b**) Height profile along the line indicated in (**a**). Reproduced with permission from [6], copyright 2007, Wiley-VCH.

AFM can be used to estimate the bilayer thickness of PEs adsorbed onto MGs following the method described by Leporatti* et al.* [45]. De Geest and coworkers [6] estimated the thickness of DEX-HEMA MGs coated with four bilayers of (DEXS/pARG) from the AFM height profile (Figure 11) taking into account that the measured height is twice the thickness of the entire system. The average thickness obtained was 45 nm, ~11 nm per DEXS/pARG bilayer, which is significantly thicker than the values reported for biopolyelectrolyte films adsorbed onto rigid and flat substrates [46].

**Figure 12:**
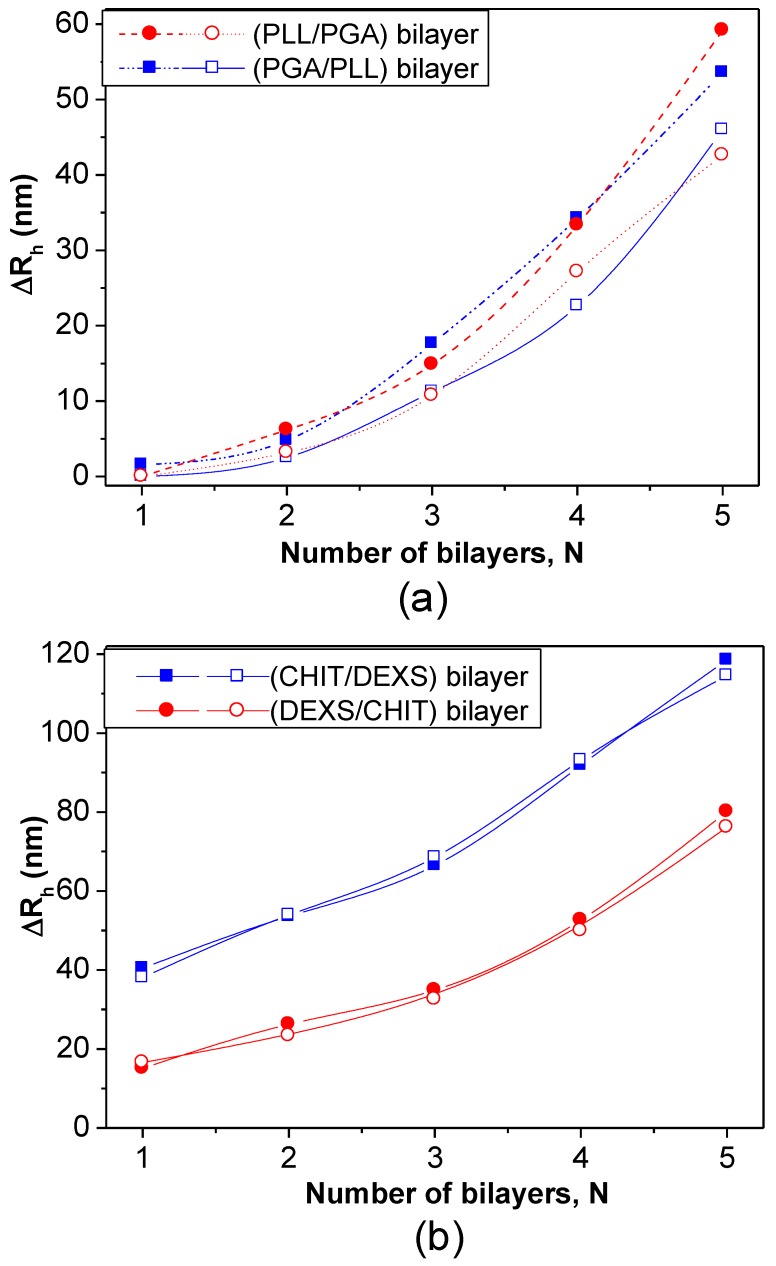
Bilayer thickness for different polyelectrolyte layers at 20 °C: (**a**) NG/(PLL/PGA) and (**b**) NG/(CHIT/DEXS). The solid and empty symbols correspond to values obtained before and after the heating-cooling cycle is applied, respectively. Reproduced with permission from [8], copyright 2010, Elsevier.

DLS measurements have also been employed to estimate the bilayer thickness of biopolymer coated PNIPAM-*co*-MMA NGs in the swollen state taking the *R*_h_ of the NG coated with the first and second layers [8]. The increment in *R*_h_ at 20 °C* vs.* the number of (PLL/PGA) and (PGA/PLL) bilayers deposited on NG/(PLL/PGA) and NG/PLL, respectively, is shown in Figure 12a. Likewise, the thickness of bilayers of CHIT/DEXS and DEXS/CHIT deposited on NG/(CHIT/DEXS) and NG/CHIT are plotted in Figure 12b. PLL- and PGA-terminated films had very similar thickness: ~3 and 55 nm for the first and last bilayers deposited, consistent with values reported for the fabrication of these biopolymers onto biological substrates [47]. For polypeptide-coated systems, values obtained from the heating curves were on average 16% higher than those derived from the cooling cycles, ascribed to the intermolecular rearrangements that polypeptides undergo when adsorbed onto NGs. In contrast, for polysaccharide-coated systems, only small differences were found between data derived from the heating and cooling curves, since their longer chains have a lower degree of freedom for reorganization. CHIT-terminated bilayers were found to be thinner than DEXS-terminated ones, indicating that the layer thickness is determined by the conformation of the biopolymer in the outermost layer. The negatively charged polysaccharide has a branched backbone and can adopt a more expanded conformation, thereby leading to thicker layers, whilst the positively charged polysaccharide displays a linear and flat structure, which therefore results in thinner layers. These bilayers are considerably thicker than those obtained upon assembly of these biopolyelectrolytes on rigid substrates [46,48], which can be rationalized by considering that the NG is in aqueous solution, and the PE layers are fully swollen and bound to a swollen substrate [8]. Surprisingly, the polysaccharide bilayers were noticeably thicker than the polypeptide ones, associated with several factors influencing the relative layer thickness, such as charge density, hydrophilicity, *M*_w_, residue size,* etc.* Despite this, polysaccharides tend to adopt a stretched conformation to minimize the repulsion between charges, which leads to thinner layers as they are more hydrophilic, adsorbing more water, leading to increased layer thickness. In addition, a raise in the polyelectrolyte, *M*_w_ and chain length usually causes an increase in the amount of material adsorbed.

As can be observed from Figure 12, both polypeptide- and polysaccharide-coated PNIPAM-*co*-MMA NGs seem to exhibit a nonlinear growth of the layer thickness with the number of deposition stages. Exponential growth was found for PLL/PGA [49] and HA/CHIT [50] multilayers assembled on solid substrates, whilst quasi linear growth was reported for PSS/PAH multilayers [51]. To explain the nonlinear buildup mechanism, different models have been proposed. McAloney* et al.* [52] suggested that such behaviour is caused by the increase in the film roughness with increasing number of deposited layers. Another viable explanation could be the ability of at least one PE to diffuse “in” and “out” of the whole structure during each bilayer deposition [46,53,54].

Other authors estimated the PE thickness using fluorescently labelled species, in particular PLL^FTIC^. Thus, the effect of dipping time on HA MGs coated with (PLL/HA)_4_-PLL^FITC^ was investigated [37]. As the adsorption time increased, the thickness of the band corresponding to PLL^FITC^ increased remarkably. Further, by keeping the adsorption time constant, it was found that the film thickness grew strongly with the number of layers, from 19.8 μm for a (PLL/HA)_4_-PLL^FITC^ to 35.8 μm for a (PLL/HA)_9_-PLL^FITC^.

### 6.6. Temporal Stability

**Figure 13:**
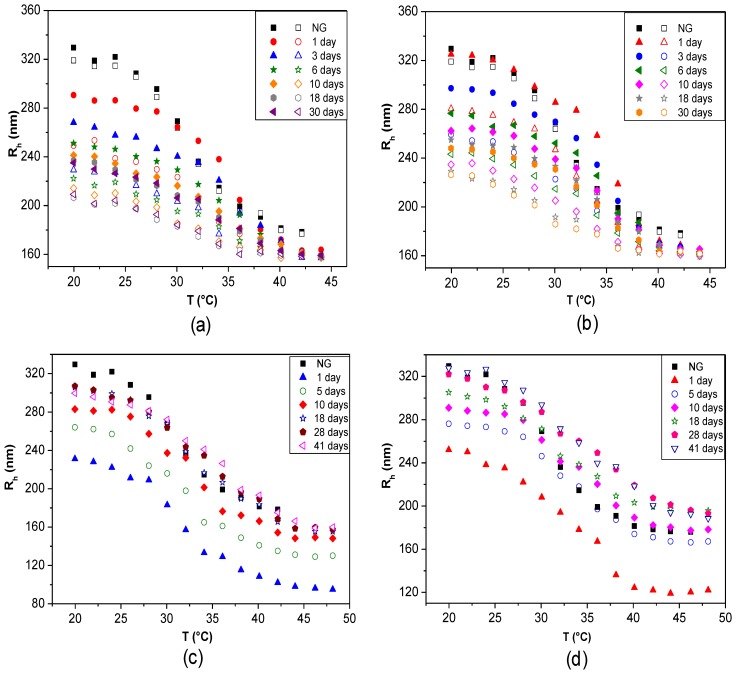
Temporal stability of LbL-coated PNIPAM-*co*-MMA NGs in water over different periods of time: (**a**) NG/PLL; (**b**) NG/(PLL/PGA); (**c**) NG/CHIT and (**d**) NG/(CHIT/DEXS). Closed and empty symbols correspond to heating and cooling cycles, respectively. Gels coated with polysaccharide layers (**c** and **d**) are reversibly thermoresponsive; for clarity, only the heating cycles are shown. Reproduced with permission from [8], copyright 2010, Elsevier.

The changes in *R*_h_ (or *D*_h_) and in the thermoresponsive behaviour as a function of time can be monitored to analyse the storage or temporal stability of LbL-coated NGs. Studies with fluorescently labelled PLL demonstrated that there is no desorption with time even after three months, corroborating that biopolymer-coated NGs are very stable [42]. DLS curves over different periods of time for PNIPAM-*co*-MMA NGs coated with: (a) PLL, (b) PLL/PGA, (c) CHIT and (d) CHIT/DEXS are shown in Figure 13 [8]. In the case of PLL-coated NG, a noteworthy decrease in *R*_h_ with time was found, by about 9%, 15%, 17%, 19% and 21% after 3, 6, 10, 18 and 30 days, respectively (Figure 13a). In contrast, when the coated NGs were heated, they always collapsed to nearly the same size as when they were first coated. Analogous trends were found for *R*_h_ of systems where the outermost layer was PGA (Figure 13b), consistent with the results reported by Pelsöczi* et al.* [47], who found a remarkable drop in the average roughness of one-month-old (PLL/PGA) films. Significant hysteresis was found between the heating and cooling cycles, as well as variations in the VPTT values, hinting towards spatial and temporal reorganization over long periods of time. Nevertheless, the differences between data obtained from the heating and cooling cycles decreased with increasing time (from ~14% to 8% over a period of one month), indicating that the NG-polypeptide interaction finally stabilizes, and it can be expected than a quasi-reversible steady state will be reached over a longer period. Consequently, it was concluded that the final conformations adopted by the polypeptides adsorbed onto the NG, caused by its thermoresponsive behaviour, are reversible. Conversely, polysaccharide-coated NGs displayed an opposite behaviour: no hysteresis was detected and *R*_h_ rose progressively with time to approximately the initial size of the raw NG. In the case of CHIT-terminated NG (Figure 13c), it rises by ~15%, 23%, 30%, 33% and 34% after 3, 6, 10, 18 and 30 days, respectively. Similar behaviour can be observed for DEXS-terminated systems (Figure 13d). The NG-polysaccharide interaction likely stabilizes these systems, and after 20 days they reach almost the same size as when first coated. The discrepancies between polysaccharide and polypeptide-coated NGs were attributed to their different level of swelling. After a long period of time polysaccharide-terminated systems, which are more hydrophilic, can absorb more water, resulting in greater disentanglement of the PE chains and the NG network, thereby allowing the whole ensemble to adopt a more expanded configuration.

De Geest* et al.* [25] subjected LbL coated DEX-HEMA MGs to changes in pH to investigate their stability. Self-rupture did not occur at pH 7.4 but at pH 9, ascribed to the pH dependent permeability of the coating. Thus, at pH 7.4 the multilayers assembled were permeable to the degradation products of the MGs whilst at pH 9 they were impermeable, hence the products remained inside the ensemble and the increase in osmotic pressure caused the system to burst.

### 6.7. Confocal Microscopy and Fluorescence Correlation Spectroscopy

**Figure 14:**
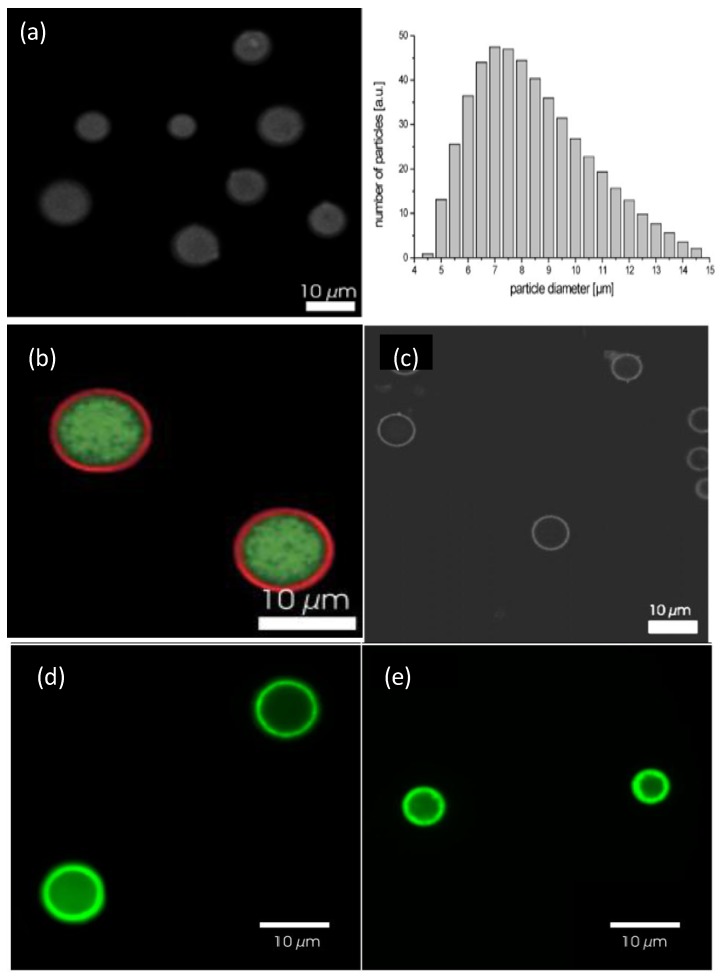
(**a**) Confocal microscopy images of uncoated DEX-HEMA MGs and their size distribution; (**b** and **c**) DEX-HEMA MGs coated with four biopolyelectrolyte bilayers before (**b**) and after (**c**) degradation of the MG. Reproduced with permission from [6], copyright 2007, Wiley-VCH; (**d** and **e**) Confocal microscopy images of polypeptide coated P(NIPAM-*co*-MAA) microgels at 24 °C (**d**) and 37 °C (**e**). Reproduced with permission from [27], copyright 2012, American Chemical Society.

Additional proof of the successful LbL assembly of biopolymers onto gels can be obtained from confocal microscopy. Figure 14a shows a confocal image of bare DEX-HEMA MGs and their size distribution as determined by laser diffraction [6]. These MGs exhibit a wide size distribution, with an average diameter of 7 μm. A representative image of these MGs coated with four bilayers of (DEXS/pARG) is displayed in Figure 14b. The MGs were fluorescently labelled with FITC-DEXS (green colour), whilst pARG was labelled with rhodamine B isothiocyanate (RITC, red colour). It can be clearly observed that a ring of red fluorescence originating from the labelled pARG surrounding the MGs is present, indicative of successful LbL coating. The DEX-HEMA core was degraded after exposure of the coated MGs to a 0.1 M phosphate buffer (pH 7.4) at 37 °C for 5 days (Figure 14c) resulting in hollow capsules; the microcapsules did not rupture, but released the FITC-DEX since no significant fluorescence was detected within the microcapsules. The influence of the PE molecular weight on the behaviour of the core-shell ensemble was also investigated [6]. Systems with low *M*_w_ PGA all ruptured, while those with high *M*_w_ PGA did not all burst. This suggests that the increase in PGA molecular weight, changes the coaction between the mechanical properties and the permeability of the biopolymer coating.

P(NIPAM-*co*-MAA) coated MGs were also characterised by Costa* et al.* [27] using confocal microscopy at 24 and 37 °C (Figure 14c,d, respectively). The fluorescent labelling demonstrated that these polyions diffuse into and distribute across the MG, with more fluorescence intensity situated on the MG shell, ascribed to the higher concentration of carboxylic acid groups in that area. Another explanation for this phenomenon might be a self-limiting adsorption process that takes place as the polycation adsorbs onto the MG surface even as it diffuses into the gel, hence producing an intrinsic barrier to the diffusion of more polymer into the MG. The presence of labelled polymer within the gel implies that PEs with higher mobility can display stronger “odd-even” effects based on size and responsive behaviour, as increased mobility offers more accessibility to the acid groups throughout the MG. A gradual rise in fluorescence was detected after each polycation deposition, following a quasi-linear regime with the bilayer number.

Bysell* et al.* [10] analysed the interaction between PLL and PAA MGs (80–120 μm diameter) by confocal laser scanning microscopy. Both peptide distribution and gel de-swelling kinetics were found to be strongly influenced by the PLL molecular weight, pH and salt concentration. A slower de-swelling was observed for the PE with higher *M*_w_, related to the slower peptide diffusion. Further, at pH 4.5 the rate of microgel de-swelling was lower than at higher pH, explainable in terms of a complex coaction between the pH-dependence of both PLL and the PAA network, also influencing the volume of the latter. Additionally, the increase in the PE concentration resulted in a decreased de-swelling rate, although the peptide transport rate within the MG was higher. These effects will play an important role in the performance of peptide-containing MGs in drug delivery applications, particularly in release rates and loading capacity.

**Figure 15:**
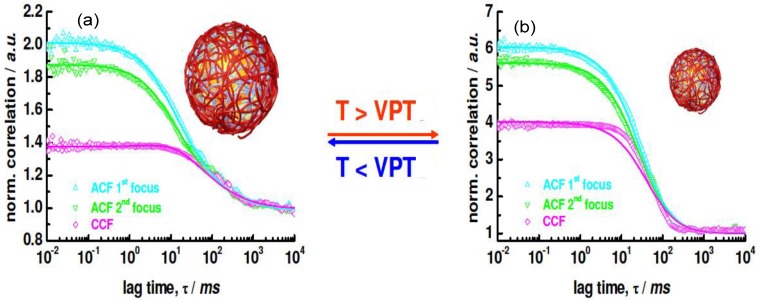
2*f*-FCS curves of uncoated rhodamine labeled NG detected at two different foci (ACF1 and ACF2) and cross-correlated (CCF) at (**a**) 25 °C and (**b**) 40 °C. Reproduced with permission from [42], copyright 2009, American Chemical Society.

With regard to NGs, it is very difficult to visualize the exact location of the LbL coating using fluorescently-labelled species. FCS has been employed to demonstrate the successful assembly of biopolymers onto PNIPAM NGs [42]. The 2*f*-FCS curves of the uncoated NG labelled with Rhodamine at (a) 25 °C and (b) 40 °C are shown in Figure 15. *R*_h_ values of the swollen and collapsed state were estimated as 276 and 174 nm, respectively, using the particle size effect model, values that are in good agreement with the *R*_h_ derived from DLS curves, which were 265 and 168 nm, respectively.

When a fluorescently-labelled biopolymer PLL^FITC^ was incorporated into the multilayer system, single or auto-correlated experiments carried out on each species at different layering stages provided similar curves [42], indicating diffusion times of the same order of magnitude, thus corroborating the anchoring of the labelled PE to the NG. If the species were moving independently, the cross-correlation curve would have shown only a baseline. Images of the dried PNIPAM/PLL^FITC^ clearly showed that the fluorescence originated exactly from the same species, demonstrating that the PLL was bound to the gel.

### 6.8. Mechanical Properties

**Figure 16:**
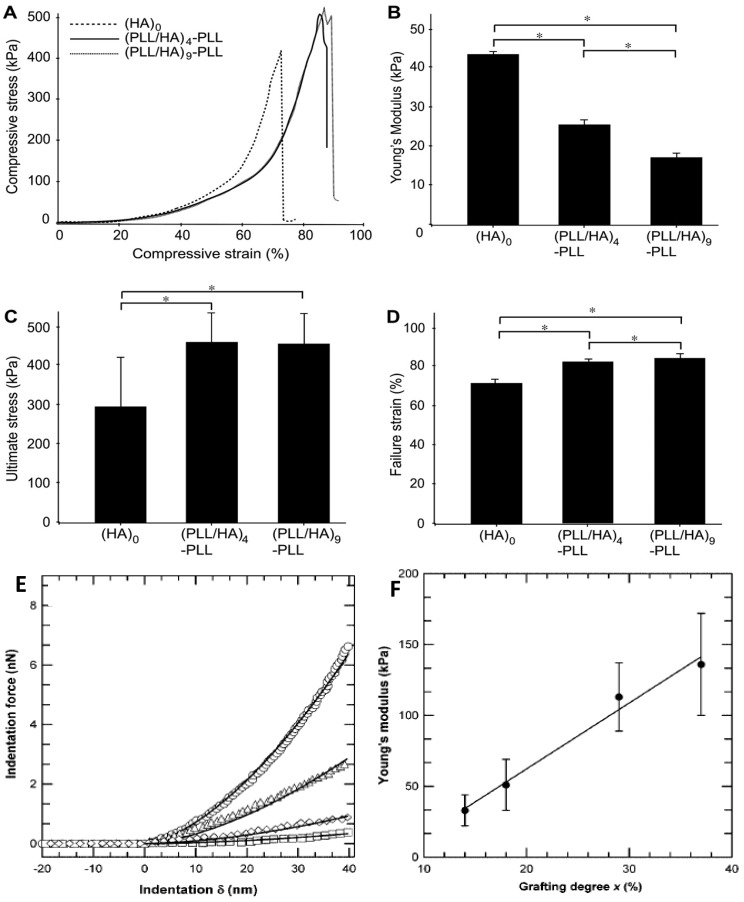
Mechanical properties of unmodified and PE coated HA MGs: (**A**) Compressive stress as a function of the strain; (**B**) Young’s modulus; (**C**) ultimate stress and (**D**) ultimate strain. Reproduced with permission from [37], copyright 2011, Elsevier; (**E**) Comparison of force* vs.* deformation curves measured by AFM for VB-*g*-HA/PLL films with grafting degrees of 14% (squares), 18% (tilted squares), 29% (triangles), and 37% (circles); (**F**) Variation of Young’s modulus extracted from force-indentation profiles of VB-*g*-HA/PLL* vs.* the grafting degree. Reproduced with permission from [29], copyright 2009, American Chemical Society.

Recently, designing PEM with adjustable mechanical properties has become a key challenge from an application viewpoint. A typical method to measure the mechanical properties is to perform nano-indentation experiments using AFM technique, frequently using a colloidal probe as indenter. Several other methods [55] have been employed to characterize the thin films including quartz crystal microbalance, piezo-rheometry, the pendant drop, the capillary wave technique, as well as tensile and compression tests. Yamanlar* et al.* [37] investigated the mechanical properties of pure HA MGs and those coated with (PLL/HA)_4_-PLL and (PLL/HA)_9_-PLL multilayers via compression tests (Figure 16).

All the systems showed similar stress-strain behaviour irrespective of the number of layer deposited (Figure 16a). However, the LbL process led to a noticeable decrease in the Young’s modulus (Figure 16b) whilst increasing the ultimate stress and strain to failure (Figure 16c,d, respectively). This behaviour was ascribed to the presence of PLL in the outermost layer that caused certain charge shielding, thus decreasing the chain repulsions and resulting in elastic behaviour at low compressive strain. However, at higher strains the higher network density due to the MG shrinking likely led to higher compressive strength. Overall, the stress-strain behaviour was similar for both coated and uncoated systems, suggesting that the observed changes in the mechanical properties were only related to the surface modification of the MGs without affecting their bulk properties, which was confirmed by AFM nano-indentation experiments.

The mechanical properties of VB-*g*-HA/PLL films were analysed by Pozos-Vazquez* et al.* [29] using AFM nano-indentation. The comparison of force-indentation curves for samples with grafting degrees from 14% to 37% (Figure 16e) revealed that the stiffness of the multilayers increased with the grafting degree. Young’s modulus data were estimated using the Hertz sphere model (Figure 16f), and it was found that the modulus grew linearly with the level of grafting, attributable to the increase in the degree of crosslinking, since the rigidity of a network is inversely proportional to the average length of the segments between two crosslinks, hence proportional to the number of VB groups grafted onto HA chains. Therefore, the stiffness of the film can be precisely tuned by simply varying the grafting degree of VB-modified HA chains incorporated into the films.

## 7. Potential Applications

Biopolymer-coated MGs (or NGs) are highly suitable candidates for a variety of biomedical applications due to the combination of their colloidal nature (e.g., colloidal stability, high surface area) with the inherent features of the macroscopic hydrogels. For instance, in the field of implantable biomaterials, they can be used for bone, vascular, dental, neuronal, and pancreas tissue engineering as well as tracheal prostheses. Scaffolds are typically produced using natural MGs like CHIT or HA, since they are porous, biocompatible and hydrophilic. They should provide enough mechanical support and allow the transport of both nutrients and growth factors to encapsulated cells. Thus, HA-based MGs, which are non-antigenic, non-inflammatory, non-pyrogenic, non-migratory, easy to use and stable after injection, long-lasting but re-absorbable, have been used for soft tissue augmentation (*i.e.*, lip augmentation and re-contouring) [56]. They have also been employed for cosmetic intradermal implants or for the treatment of vocal fold insufficiency. Moreover, they can act as a matrix for fibroblast growth factor delivery in repair of bone fractures. On the other hand, thermoresponsive HA-*g*-PNIPAAM microgels were shown to serve as pharmaceutical delivery vehicles for a variety of ophthalmic applications [57] and for adipose tissue engineering [58]. PNIPAM-*co*-HEMA as well as PNIPAM-*co*-AA have been used as 3D cell scaffolds [59].

Another interesting application of biopolyelectrolyte/MG systems is gene delivery, that is, the process of introducing exogenous genes into host cells and the translation of the information carried by those genes into functional proteins. Gene delivery has potential applications in the treatment of different diseases including cancer and can be used in the field of vaccination. The controlled delivery of DNA complexes from LbL coated ensembles offers the opportunity to enhance gene transfer by maintaining an elevated concentration of DNA within the cellular microenvironment using an appropriate polyelectrolyte film carrier that facilitates DNA introduction. In this regard, biodegradable microcapsules made of DEXS/pArg [60] as well as HA-MG scaffolds [61] have been used as gene delivery vectors.

Biosensors based on responsive MGs have also been reported. For instance, different core-shell PNIPAM-*co*-AA MGs incorporating 3-aminophenylboronic acid (APBA), a common probe for the detection of glucose, have been developed and the glucose-induced swelling was found to be dependent on the MG structure [62]. Further, the immobilization of biomolecules on the surface of MG systems has been employed for sensing applications. Thus, acrylic MGs have been used in amperometric sensors as immobilizing support for different enzymes like glucose oxidase and tyrosinase [63]; similarly, amperometric sensors using tyrosinase entrapped in PNIPAM MGs have been developed for determination of phenolic compounds [64]. Experimental results have shown that the MG structure has a great influence on the sensor response; hence its sensitivity and selectivity can be tuned by modifying the gel characteristics (*i.e.*, hydrodynamic size, degree of crosslinking) as well as the nature of the absorbed PEs and their number of layers.

The most important biomedical application of LbL-coated MGs is as drug delivery carriers. Their tuneable size, large surface area, porous nature and swelling ability make them ideal for encapsulation and controlled release of bioactive substances including peptides, proteins, enzymes, DNA, drugs and cells, since they provide a biomimetic* in vivo* aqueous environment where peptides or enzymes are prevented from drying. The pH, ionic strength, and temperature are the most useful triggers for controlling the shrinking behaviour of MGs. Several studies have reported the use of PNIPAM microgels as drug carriers because they have a LCST close to body temperature [65,66]. According to a “squeezing out” mechanism, the drug remains in the swollen MG below the LCST and it is released from the shrunken MG upon heating. Recently, multifunctional scaffolds based on PNIPAM-*co*-AA MGs coated with poly(lactic-*co*-glycolic acid) (PLGA) biopolymer were developed as carriers of vancomycin, which is a suitable drug that imparts antibiotic function to the scaffolds [67]. On the other hand, polysaccharide-based MGs (or NGs) that have high water content, are renewable, nontoxic, and relatively cheap have great potential for use in drug delivery. In this regard, a pulsed drug delivery system was prepared by LbL coating pH-sensitive CHIT-ALG MGs with PSS and PAH [68]. The drug release of this novel system was governed by the swelling pressure of the core and the rupture of the outer membrane. Further, it is possible to control the mechanical properties and the permeability of the PE shell by tuning the layer thickness and density on the nanometre scale, thereby enabling the loading of different biomolecules such as peptides, proteins, and oligonucleotides.

## 8. Concluding Remarks and Future Outlook

This review focuses on the preparation and characterization of biopolymer-coated thermo/pH-responsive micro/nano-gels like synthetic PNIPAM and PAA or natural HA via the LbL technique, analysing the influence of the polyelectrolyte nature and the assembly conditions on the behaviour of the coated templates. The swelling and de-swelling processes are nearly reversible for polysaccharide-terminated gels; in contrast, templates coated with polypeptides display strong hysteresis owing to temperature triggered conformational changes induced by adsorption onto the gel. Moreover, an “odd-even” effect on the size of the systems has been detected depending on the nature of the polyelectrolyte in the furthest layer. Literature data corroborate that with the appropriate selection of the biopolymers, the morphology and responsivity of the coated gels can be finely tuned and their temporal stability enhanced, making them highly suitable for a broad range of biomedical applications, such as, biosensors, gene delivery as well as encapsulation (storage) and release of small molecules or drugs. Further, coated MGs are ideal candidates for application in the field of tissue engineering. In the future, biomaterials that better mimic the natural extracellular matrix in terms of composition, structural characteristics, and mechanical properties are likely to be employed. In this regard, ongoing research should focus on the combination of synthetic MGs that provide mechanical tailoring possibilities with biopolyelectrolytes like glycosaminoglycans or polypeptides that can mimic the extracellular matrix to a greater extent. On the other hand, the ability of coated ensembles to release substances in a controlled range of pH and temperature may serve as basis for the development of new means of* in vivo* delivery of proteins, enzymes or vaccines. Future challenges include the use of biodegradable gels and shells like biopolymer-coated DEX-HEMA microgels that are biocompatible, the efficient loading and release of the drug, the incorporation of new functional groups to obtain site-specific target delivery and the addition of inorganic nanoparticles to develop hybrid core-shell structures with enhanced optical, magnetic and electronic properties. Nevertheless, research on biomedical applications of these types of systems is still in its infancy, and is only for laboratory use. Their performance* in vivo* needs testing/investigating and clinical results are lacking. A lot of research on this topic is required prior to cost-effective large-scale use.
